# Diagnostic Value of Nonacid Nucleic Blood Tumor Marker Panels in Early Diagnosing Breast Cancer: A Systematic Review and Network Meta-Analysis

**DOI:** 10.1155/2022/4119345

**Published:** 2022-02-03

**Authors:** Vahid Raja, Ziba Farajzadegan, Marjan Mansourian, Khojaste Ghasemi, Mohammad Sadegh Aboutalebi, Rasool Nouri, Fariborz Mokarian

**Affiliations:** ^1^Clinical Laboratory Sciences, Amin Hospital, Isfahan University of Medical Sciences, Isfahan, Iran; ^2^Community & Preventive Medicine, Isfahan University of Medical Sciences, Isfahan, Iran; ^3^Biostatistics, Department of Biostatistics and Epidemiology, School of Public Health, Isfahan University of Medical Sciences, Isfahan, Iran; ^4^Department of Biostatistics and Epidemiology, School of Public Health, Isfahan University of Medical Sciences, Isfahan, Iran; ^5^Faculty of Nursing and Midwifery, Isfahan University of Medical Sciences, Isfahan, Iran; ^6^Department of Medical Library and Information Sciences, Isfahan University of Medical Sciences, Isfahan, Iran; ^7^Hematology & Oncology Department of Internal Medicine, Isfahan University of Medical Sciences, Isfahan, Iran

## Abstract

This study is aimed at determining the best nonacid nucleic blood tumor marker panels in terms of sensitivity, specificity, and accuracy in order to detect breast cancer in early stages (I, II, and III) among eligible women for breast cancer screening. PubMed, Web of Science, Embase, Scopus, and Cochrane were systematically reviewed to assess nonacid nucleic blood tumor marker panels' diagnostic value in women, both healthy and patient (before any anticancer treatment), for detecting breast cancer. A network meta-analysis was carried out using a Bayesian network meta-analysis to estimate combined odd ratio (OR) and 95% CI credible interval for presenting the results. Rankograms plot was drawn to rank the diagnostic value of different panels. Of the 2358 titles initially identified, 9 studies and 8 panels were included in the network meta-analysis. Panels A (MMP-9/TIMP-1) and K (TF1+TF2+TF3) had the highest sensitivity in early stages, as panel A with OR = 11.61 and 95% CI (1.49-102.5) demonstrated a better function than mammography. Panels H (CA 15.3 + IL-18) and A (MMP-9/TIMP-1) had the highest specificity in early stages, but no significant difference with mammography. Panels A (MMP-9/TIMP-1) and H (CA 15.3 + IL-18) had the highest accuracy in early stages, as they significantly exhibited a higher function than mammography with OR = 6.87 and 95% CI (2.07-31.35) as well as OR = 3.44 and 95% CI (1.15-11.07), respectively. Panel A including MMP-9/TIMP-1 in early stages demonstrated a higher diagnostic value for breast cancer than the rest of the panels.

## 1. Introduction

The cancer is the second prevalent cause of mortality after cardiovascular diseases in the world and developed countries, and the third cause in developing countries [1]. Among the different types of cancers, breast cancer includes 23 percent of all the women cancers and is the most prevalent cancer as well as the deadliest malignancy among women. It is also the second cause of death which is resulted from the cancer after the lung cancer and one of the most important worryingly causes of women's health [2–4]. Although breast cancer is the most prevalent cause of cancer death among women, the early diagnosis can raise chance of treatment and complete remission [5] Therefore, it has an essential role in the type and efficacy of treatment [6–8].

Based on the European Commission Initiative on Breast Cancer (ECIBC), screening should be followed by regular mammography and in combination with annual MRI for women with familial breast cancer [9].

According to the International Agency for Research on Cancer (IARC), World Health Organization (WHO), and eighth edition of American Joint Committee on Cancer (AJCC), diagnostic work-up for early breast cancer can be divided into four assessments that are as follows: general health status such as history taking, physical examination (CBE and BSE) and full blood count, assessment of primary tumor including physical examination in combination with imaging methods along with core biopsy, examination of regional lymph nodes, and detecting of metastatic disease [9].

According to the cited guideline, the prevalent methods of screening and diagnosing breast cancer include imaging methods (mammography, MRI, and breast ultrasound), physical examination (CBE: clinical breast exam; BSE: breast self-exam), biopsy, and using blood factors, among which mammography is a common and gold standard method for detecting breast cancer [5, 10–12]. Nevertheless, because of the limitation of these methods, particularly mammography [5, 11, 13–24], cancer is usually detected when tumorous cells rise locally and invade the surrounding tissues, axillary lymph nodes, and even other parts of the human body. As a result, clinical symptoms and pathological manifestations become visible.

Late diagnosis of the cancer is one of the most important reasons of patients' death [25, 26]. In addition, we usually need more time and money on verifying the diagnosis since some of the diagnosis may be incorrect and unreliable [11, 27, 28].

Generally, we need more investigation and evaluation to determine a simple, accessible, cost-effective, and reliable method for screening breast cancer in early stages.

Among the different methods of breast cancer diagnosing, using blood factors (tumor markers) is known as a simple, noninvasive, and accessible method [29]. However, it is not used as a common and standard method for breast cancer screening because of low sensitivity and specificity. As a result, this method is predominantly used for monitoring treatment, determining recurrences, and evaluating malignancy progress [30–33].

There are some studies, in which several blood factors (tumor markers) are simultaneously evaluated as a panel that comparatively have higher sensitivity and specificity in diagnosing breast cancer than the individual state. Therefore, a single panel of blood tumor markers with high sensitivity and specificity can be promising for early diagnosing of breast cancer.

We decided to include non-acid nucleic blood tumor marker panels for carrying out a network meta-analysis because they are comparatively more cost-effective than acid nucleic blood tumor markers and can be easily utilized and set up in different clinical laboratories with various levels of facilities and instruments [29].

This study is aimed at preparing new knowledge about the diagnostic value of the best panels of nonacid nucleic blood tumor markers with different components to detect breast cancer, especially in early stages, by carrying out a network meta-analysis.

## 2. Material and Method

The systematic reviews of the observational studies were conducted based on PRISMA guidelines (Preferred Reporting Items for Systematic Reviews and Meta-analysis) [34].

### 2.1. Eligibility Criteria

Definition of PICO:

Participants: qualified women for screening breast cancer

Intervention: nonacid nucleic blood tumor markers panels

Comparison: gold standard test

Outcome: sensitivity and specificity

(1) Studies that have investigated the chemical tumor markers called as nonacid nucleic in breast cancer, which are measurable in peripheral blood; (2) studies that have simultaneously assessed several tumor markers in the form of a panel to diagnose and detect breast cancer in early stages (I, II, and III); (3) articles presenting data for estimating sensitivity and specificity; (4) having the minimum of two and maximum of five as the number of tumor markers in the panel; (5) all cohort and case control studies, in which accuracy in healthy and nonhealthy (before any anticancer treatment) people is compared; (6) studies being published in English.

### 2.2. Identifying Relevant Evidence

#### 2.2.1. Search Strategy and Databases

PubMed, Web of Science, Embase, Scopus, and Cochrane collaboration were searched for identifying the related studies (Appendix B) ((PubMed up to 28 October 2019), (Web of Science + Scopus + Embase up to 27 November 2019), and (Cochrane up to 3 December 2019)).

The following key words as well as MESH terms (medical subject heading) in PubMed were used to find the related articles:

1. Search (“breast cancer mucin” OR “cancer antigen27.29”OR cancer antigen15.3 OR “cancer antigen125”OR “cancer antigen549”OR “cancer associated serum antigen” OR “catapsin d” OR “carcinoma embryonic antigen” OR “creatine kinase-brain” OR “carboxypeptidase n” OR “collapsin response mediator proteins” OR “colony stimulating factor1” OR “cytokeratin fragment” OR “Galectin.3” OR “human epidermal growth factor receptor-2” OR “Human mammaglobin” OR “mannose receptor” OR “mucin- like carcinoma” OR “Matrix metalloproteinase-9” OR “Mammary serum antigen” OR “Nicotinamide phosphoribosyl transferase” OR “p53 protein” OR “phosphohexose isomerase” OR “small breast epithelial mucin” OR Survivin OR “Tumor-associated glycoprotein72” OR “Tissue Inhibitor of Metalloproteinases-1” OR “Thymidine kinase” OR “Tissue polypeptide antigen” OR “Tissue polypeptide-specific antigen” OR “Urokinase plasminogen activator” OR “plasminogen activator inhibitor-1” OR “Vascular Endothelial Growth Factor” OR “E_selectin:endothelial selectin” OR “P-selectin:platelet selectin” OR “intracellular adhesion molecule 1”)
2. Search “Biomarkers, Tumor/blood”[Mesh)

Search ((((#1 OR #2))) AND ((“Early Detection of Cancer”[Mesh]) OR ((“Cancer Screening” OR “Cancer diagnosis”)))) AND ((“Breast Neoplasms”[Mesh]) OR “breast cancer”).

#### 2.2.2. Study Selection and Data Extraction

All the considered publications were screened for relevance by two independent reviewers, and any disagreement on the title and abstract of the studies was dissolved by discussion with the third person as a principal investigator. The full texts of the relevant studies were checked based on the inclusion criteria by researchers. The final list of the eligible studies was prepared after reaching a consensus between the two researchers. The extracted data from all the eligible studies were as follows: study design, year of publication, location, first author's last name, study sample size, type of panels, number of panel components, sensitivity, specificity, accuracy of panels, method of chemical measurement, type of samples, clinical stages, outcome definition, and risk of bias (Appendix ATable 1). Qualitative evaluation of individual articles was independently assessed by two researchers, and the scoring system based on the CASP checklist (specified for diagnostic studies) was applied. The objective of the study, sources and measurements, statistical methods, results in data, main results, and study limitation were evaluated. Each of the 12 questions in the checklist was scored as 0, 0.5, or 1 (yes: 1; cannot tell: 0.5, no: 0). Based on these mean scores, the quality of studies was categorized into three groups of high (acquired 70 percent of the total score), moderate (acquired 50-69 percent of the total score), and low quality (under 50 percent of the total score). Overall, 94.4 percent of studies had high quality, and other studies were of moderate quality.

The included panels were as follows: (A: MMP-9/TIMP-1, B: M-CSF+CA15-3, C: VEGF + CA15-3, D: VEGF + M-CSF+CA 15-3, E: VEGF+ M-CSF, H: CA 15.3+IL-18, I: MSA+B2m, and K: TF1+TF2+TF3).

All these panels were made based on simultaneously measuring two or three blood tumor markers in the patient and healthy people using a compatible linear combination method [35], except panel A that was resulted from the ratio of two tumor markers (MMP-9 and TIMP-1). Measuring protease (MMP-9)/protease inhibitor (TIMP-1) ratio in some tumors may be a more accurate reflection of ECM (extracellular matrix) remolding than measuring the absolute level of the corresponding protease. Accordingly, authors measured the MMP-9/TIMP-1 ratio, and it was higher in the malignant group than benign and control groups [36]. Panels B, C, D, and E were assessed in more than one study (multiple studies), and panels A, H, I, and K were only assessed in one study (single study).

We conducted direct and indirect paired comparisons of the sensitivity, specificity, and accuracy of the included blood tumor marker panels for screening breast cancer in early stages. All the investigations were conducted in comparison to mammography (M) method as the gold standard [37–39].

#### 2.2.3. Statistical Analysis

Two main steps were taken to analyze the current paper: first, the traditional pairwise meta-analysis and then the network meta-analysis. In the first step, sensitivity, specificity, and accuracy were compared with the estimated odds ratios (ORs) and 95% confidence intervals (CI). Also, *I*-square and Tau-square tests were run to detect the amount of heterogeneity for each pairwise meta-analysis [40, 41]. In the second step, to clarify direct and indirect comparisons, a network plot of all the diagnostic panels was depicted. The size of nods and lines in the network plot showed the number of patients and involved studies, respectively, for each direct comparison. Pooled effective sizes were estimated by using Bayesian network meta-analysis for all the direct and indirect comparisons. The Bayesian analysis used samples of Markov chain generating by Monte Carlo simulation by noninformative priors for both effect sizes and precision. Convergence was checked and confirmed after four chains and a 10,000-simulation burn-in phase. Finally, direct probabilities were resulted from the additional 50,000-simulation phase [42, 43]. The estimates ORs and 95% credible interval (CrI) were considered for presenting the results; the ones not containing 1 were considered statistically significant. *I*^2^ statistics was calculated to assess the heterogeneity by using the Markov chain Monte Carlo (MCMC), and *I*^2^ > 50% represented high heterogeneity among the same comparative groups [44, 45]. To assess the assumption of consistency among direct and indirect evidence, the node-splitting method was applied [46, 47]. Rankograms were used to diagnose panel's performance for each modality. Every panel contained several bars that showed cumulative probability from the first [48] to the last rank (worst) [49, 50]. All the calculations were performed by R-4.0.3 using “gemtc” and “netmeta” packages for network meta-analysis and “meta” package for traditional meta-analysis [51–53].

## 3. Result

### 3.1. Study Selection

Of the 2358 retrieved articles, 479 original studies were excluded, and 475 full texts were assessed for eligibility. Fifty-four studies were identified relevant to our research question, which contained 86 unique blood tumor marker panels conforming to our eligibility criteria (Appendix DTable 1). However, we could just utilize a few number of panels in our review after expert panel discussion. Discussions' results were as follows: (1) it was approximately impossible to compare all the identified panels (86 unique panels) in the form of systematic review and network meta-analysis; (2) most of the panels were only assessed in one study (80 panels), and it was a big problem to make a network and compare panels in the systematic review and network meta-analysis. Finally, we decided to use all the multistudy panels (6 panels) and the same number of single-study panels (6 panels) (Appendix E). Among the single-study panels (80 panels), we chose the panels which had simultaneously the highest sensitivity and specificity in diagnosing breast cancer in the whole stages (I, II, III, and IV, mostly without metastasis). In addition, they must have been made of the least number of tumor markers. Overall, 15 studies and 12 panels were chosen, but only 9 studies and 8 panels presented enough data for estimating sensitivity and specificity in early stages (I, II, and III) and could be included in the systematic review and network meta-analysis. All the 9 studies were similar regarding the preanalytical procedures (Appendix F) and the analytical methods (Table 1).

**Figure 1:**
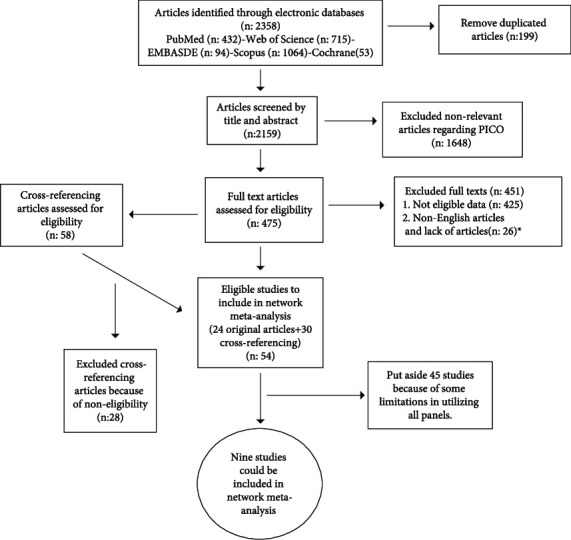
Flow diagram of included and excluded articles. ^∗^Although we sent emails to articles' authors to get their full texts, we did not receive any answers (Appendix C).

All the included and excluded studies are presented in Figure 1.

### 3.2. Study Design and Population

This study included one cohort and eight case–control studies conducted in five different countries from Asia, Australia, Africa, and Europe. Asian study was related to Japan (1 study) [54]. European studies (five works) were conducted in Poland [55–59]. Three other studies were related to Australia (1 study) [60] and Africa (2 study) [36, 61].

The publication year of studies ranged from 1987 to 2017. Sample sizes ranged from 65 [61] to 240 [59], representing the total of 1,594 participants: 857 were breast cancer, 416 healthy, and 321 participants were as benign breast tumor. Indeed, two studies had no benign breast tumor category [54, 61]. There were 704 patients (82.1%) in stages I, II, and III and 153 (17.9%) in stage IV of cancer totally. The mean age of the women in the selected studies (46.57 ± 7.12) ranged from 17 to 88 years (except the study, in which the age of population was not mentioned) [60]. The main characteristics of all the 9 studies included in the meta-analysis are shown in Table 1.

Association between diagnosing breast cancer and blood tumor marker panels in early stages: traditional pairwise meta-analysis was conducted to estimate OR and 95% CI for sensitivity, specificity, and accuracy. The results are reported in (Appendix ATable 2). Analysis of heterogeneity was conducted for three diagnostic modalities in early stages. In the early stages for sensitivity, *I*^2^ was 77%. Also, for specificity, *I*^2^ was 68%. Results represented the heterogeneity among all the comparison groups. Accordingly, the Bayesian network meta-analysis in the format of random effect model was run. However, the fixed effect model was used for accuracy because *I*^2^ was 0% for the early stages, which meant no evidence of heterogeneity among the studies. To assess the inconsistency between the indirect and direct comparisons for all the modalities, the node-splitting analysis was done; the result did not show significant inconsistency (*p* value > 0.05) (Appendix A Tables 3–5).

**Figure 2:**
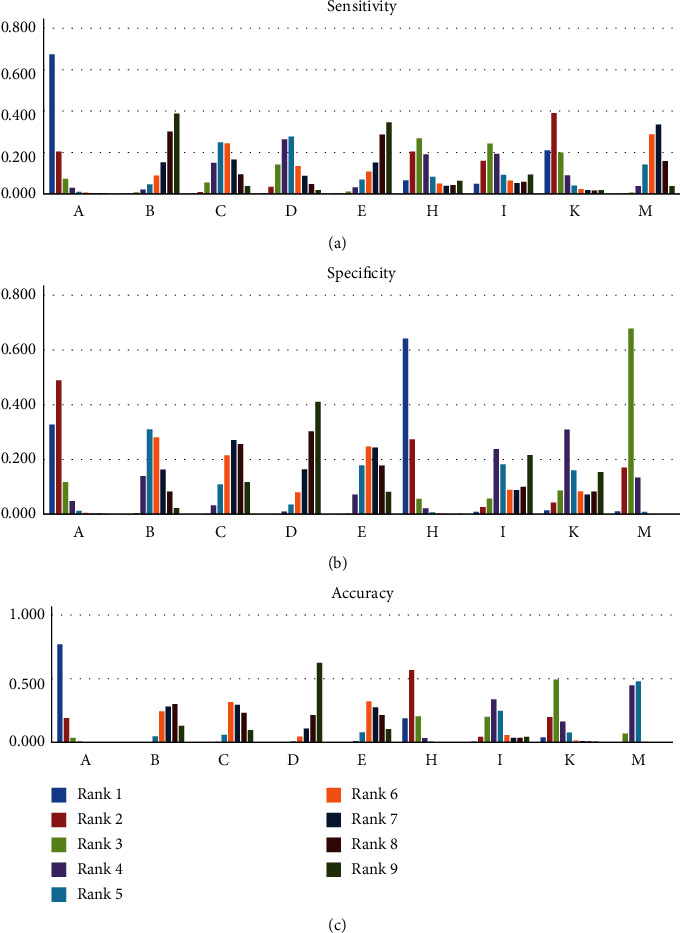
Estimated rank probability of all panels' sensitivity, specificity, and accuracy in early stages. A: MMP-9/TIMP-1; B: M-CSF+CA15; C: VEGF + CA 15-3; D: VEGF + M-CSF + CA 15-3; E: VEGF+ M-CSF; H: CA 15.3 + IL-18; I: MSA + B2m; K: TF1+TF2+TF3; M: mammography.

**Figure 3:**
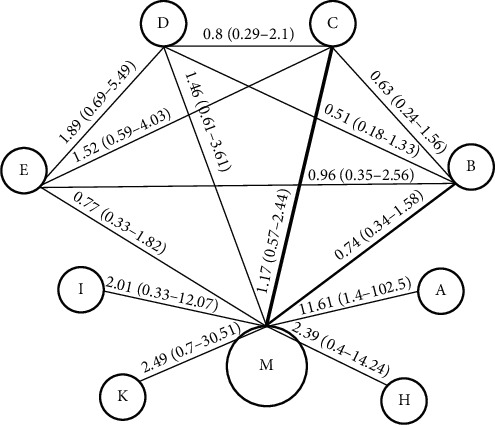
Multiple comparison of different panels for sensitivity in early stages. A: MMP-9/TIMP-1; B: M-CSF+CA15; C: VEGF + CA 15-3; D: VEGF + M-CSF + CA 15-3; E: VEGF+ M-CSF; H: CA 15.3 + IL-18; I: MSA + B2m; K: TF1+TF2+TF3; M: mammography.

Panel A had a significantly better function than M in sensitivity with OR = 11.61 and 95%CL (1.49-102.5). Panels C, D, H, I, and K exhibited a negligibly better function than M. Finally, M had a negligibly better function than E and B in sensitivity. Panel A remarkably demonstrated a better function than panels B with OR = 15.5 and 95%CL (1.8-159.59), E with OR = 14.87, 95%CL (1.65-154.2), and C with OR = 9.88, 95%CL (1.09-93.44). Also, panel A had a negligibly higher function than other panels. It had the maximum difference with panel B and minimum difference with panel K. There was no significant difference between the rest of the panels (Table 2). The highest function in sensitivity was related to panel A that was located in rank 1 with the probability of 0.674 and panel K that was located in rank 2 with the probability of 0.390. The lowest function was related to panel B that was located in rank 9 with the probability of 0.388 and panel E that was located in rank 8 with the probability of 0.286 (Figure 2(a) and Table 3). The network meta-analysis plot of the panels was drawn for sensitivity (Figure 3). M had a noticeably better function than panels B with OR = 0.15 and 95%CL (0.05-0.4), C with OR = 0.1, 95%CL (0.03-0.26), and D with OR = 0.07, 95%CL (0.02-0.21) in specificity. It exhibited a negligibly higher function than panels I and K. Panels H and A had a negligibly better function than M in specificity. Panel A exhibited a significantly higher function than panels B with OR = 19.94 and 95%CL (1.63-293.1), C with OR = 30.24, 95%CL (2.58-414.3), and D with OR = 43.87, 95%CL (3.68-669.7), but had no significant difference with other panels. Panel H had a considerably better function than panels B with OR = 0.03 and 95%CL (0.01-0.37), C with OR = 0.02, 95%CL (0.0-0.24), D with OR = 0.01, 95%CL (0.0-0.17), and E with OR = 0.02, 95%CL (0.0-0.29). Panel H had a higher function than other panels in specificity, which was not significant. There was no significant difference between the rest of the panels (Table 4). The highest function in specificity was related to panel H that was located in rank 1 with probability of 0.641 and panel A that was located in rank 2 with probability of 0.488. The lowest function was related to panel D that was located in rank 9 with probability of 0.410 and panel C that was located in rank 8 with probability of 0.256 (Figure 2(b) and Table 5).

M had a better function than panels B with OR = 0.53 and 95%CL (0.35-0.78), C with OR = 0.55, 95%CL (0.37-0.81), D with OR = 0.43, 95%CL (0.27-0.68), and E with OR = 0.55, 95%CL (0.35-0.88) in accuracy, which was significant. Panels A with OR = 6.87 and 95%CL (2.07-31.35) and H with OR = 3.44 and 95%CL (1.15-11.07) exhibited a remarkably higher function than M. Moreover, K and I had a better function than M in accuracy, which was negligible. Panel A demonstrated a significantly higher function than panels B, C, D, E, and I; it was negligibly higher than K and H. Panel H had a noticeably better function than panels B, C, D, and E, which was negligibly better than K and I. Panel K exhibited a significantly higher function than panels B, C, and D in accuracy. There was no considerable difference between the other panels (Table 6). The highest function in accuracy was related to panel A that was located in rank 1 with the probability of 0.768 and panel H that was located in rank 2 with the probability of 0.567. The lowest function was related to panel D that was located in rank 9 with the probability of 0.624 and panel C that was located in rank 8 with the probability of 0.232 (Figure 2(c) and Table 7).

## 4. Discussion

One the most important obstacles in the timely diagnosis of breast cancer is lack of a simple, accessible, precise, and reliable screening method. Among the different methods of breast cancer diagnosis, using blood factors (tumor markers) is known as a simple, noninvasive, and accessible method. However, blood tumor markers individually may not be a reliable method for breast cancer screening because of low sensitivity and specificity. On the contrary, using several blood tumor markers simultaneously as a panel can approximately promote sensitivity and specificity of single blood tumor markers in the early diagnosis and screening of breast cancer. This study is aimed at introducing the best nonacid nucleic blood tumor marker panels when coming to sensitivity, specificity, and accuracy in order to detect breast cancer in early stages. To reach this goal, we conducted a network meta-analysis and compared the best available nonacid nucleic blood tumor marker panels with mammography and each other.

Panels A (MMP-9/TIMP-1) and K (TF1+TF2+TF3) had the highest sensitivity in early stages, as panel A with OR = 11.61 and 95% CI (1.49-102.5) demonstrated a better function than mammography. It means the odds of detecting early cancer in positive individuals was 11.61 times of the odds of the outcome in people with the negative test. Nevertheless, panel K with OR = 4.59 and %95Cl (0.7-30.51) did not exhibit a significant difference with mammography. In the screening tests, sensitivity had a critical role in detecting a disease [62] since with higher sensitivity, the possibility of false negatives would be less and the time for more definitive diagnostic tests would be saved. It seems that the tumor marker panels like A or K that are able to detect breast cancer with high sensitivity in early stages can be more advantageous than mammography for screening breast cancer, especially in underdeveloped areas, because they are more accessible, cost-effective, and applicable in such regions which lack sophisticated instruments and specialized person as the two necessary factors for regular mammography.

Panels H (CA 15.3+IL-18) and A (MMP-9/TIMP-1) had the highest specificity in early stages, but did not have remarkable difference with mammography. Obviously, specificity is the proportion of people without diseases that have a negative blood test.

Panel A had 95% specificity, meaning that would identify 95 percent of healthy women who do not have the disease. Tests with high specificity (a high true-negative rate) are the most useful when the result is positive. False-positive test can impose great psychological stress on the patient or incur higher diagnostic costs.

The best panels based on total function are A: MMP-9/TIMP-1 and H: CA15.3+IL-18.

To judge a test, its sensitivity and specificity must be considered in general as a test accuracy, i.e., its ability to differentiate the patient and healthy cases correctly.

Our finding showed that in early stages, panels A and H had the highest accuracy and total function in comparison with other panels. However, we recommend panel A, because it had even better function in accuracy than panel H (Table 1). Indeed, regarding the quality of study and sample size issues, the study related to panel A was more reliable than the panel H study. Also, panel A was more cost-effective than panel H considering the price of ELISA kit. Panel A was made of MMP-9 as a member of matrix metalloproteinases (MMPs) that are implicated in cancer invasion and metastasis and TIMP-1 as a kind of tissue inhibitor of metalloproteinases (TIMPs). In the neoplastic disease, the imbalance of MMPs and TIMPs, leading to the excess of degradative activity, is supposed to be linked to the invasive character of tumor cells. In reference to MMP-9 and TIMP-1, a significant correlation has been previously reported between serum expression and breast disease severity score, suggesting the potential application of its measurement in monitoring breast cancer progression [36].

## 5. Strengths and Limitations

There are a large number of research publications about tumor markers and their correlation with monitoring the treatment, determining the recurrence, evaluating the malignancy progress, and diagnosing breast cancer. However, no systematic review and network meta-analysis have ever been reported to investigate the association between blood tumor markers panels and diagnosing breast cancer in early stages. The main strength of this study was to evaluate this idea that blood tumor marker panels can be promising diagnostic tests beside mammography for detecting breast cancer in different stages, especially the early stages. We tried to search most of the important databases; therefore, we believe we have included all the relevant studies, most of which had a high score based on the CASP checklist.

Our analysis was limited by the data in the included studies and the structure of the reported data. For example, after final screening of studies, we faced many panels (86 unique panels), all of which could not be used in our analysis. We could just use a limited number of them, as was discussed in the study selection. Most of the panels were only assessed in one study and had no more than one study (80 panels); it was a big problem to make a network and compare the panels in systematic review and network meta-analysis. We had to calculate sensitivity and specificity of panel E in the study [59] by AUC (area under the ROC curve) chart. Unfortunately, most of the studies have not exactly cited the gold standard test used for detecting patient and healthy control; therefore, we considered the gold standard test of mammography in all the studies with constant sensitivity and specificity [37–39]. Probably, some studies such as potentially non-English language studies and those lacking accessible full text were not included.

## 6. Conclusion

In conclusion, panel A including MMP-9/TIMP-1 in early stages demonstrated a higher diagnostic value for breast cancer than the rest of the panels, as it had OR = 6.87 and 95%CL (2.07-31.35) and exhibited noticeably higher accuracy than mammography as well as the highest sensitivity (97.5) among other panels and the highest specificity (95) after panel H (CA 15.3+IL-18). After panel A, panel H with sensitivity of 88.6 and specificity of 100 and panel K (TF1+TF2+TF3) with sensitivity of 90 and specificity of 93 had the best function in early diagnosing of breast cancer in comparison with the rest of the panels, which provided evidence toward further development of the early diagnosis of breast cancer in order to improve the survival of patients suffering from breast cancer. However, we definitely need more experimental studies on these panels with more sample size, in different populations and by other chemical measurement methods.

## Figures and Tables

**Table 1:** Characteristics of included articled in network meta-analysis.

First author and year	Country	Study design	Sample size and population	Clinical stages	Panel	Number of panel components	Sensitivity	Specificity	Accuracy	Method of chemical evaluation	Type of sample	Score
S Zajkowska, M. 2016 [59]	Poland	Case-control	240 Bc: 120 B: 60 H: 60 Median age (range) 54 (34–72)	I: 29 II: 30 III: 31 IV: 30	VEGF + CA 15-3 M-CSF + CA 15-3 VEGF + M-CSF + CA 15-3 VEGF+ M-CSF^∗^	2 2 3 2 VEGF M-CSF CA 15-3	95 (E) 88.3 (E) 95 (E) 87 (E) 76.25 60 83.75	65 67.5 57.5 76 85 90 75	77.8 (E) 76.4 (E) 73.5 (E) 80.7 (E)	ELISA CMIA	Plasma	11
Ikemura, M. 2017 [54]	Japan	Cohort	178 **Bc: 94** H**: 84** 56 ± 12.5 26-88	I: 42 II: 14 III: 20	TF1+TF2+TF3 TF1 TF2 TF3	3	90 67 78 87	93 68 79 48	91.4	ELISA	Serum	11
Swellam, M. 2014 [36]	Egypt	Case-control	160 **Bc80** **B40** **H40** **35.6(17-67)**	I: 46 III/II: 34	MMP-9/TIMP-1 (ratio)	2 MMP-9 TIMP-1	97.5 97.5 95	95 95 92.5	96.25	ELISA	Serum	11
Ławicki, Sławomir 2016 [57]	Poland	Case-control	200 **Bc100** **B50** **H50** **48 (20-78)**	I/II/III: 77 IV: 23 (with metastases)	VEGF +CA15-3	2 VEGF CA 15-3	76 (E) 61 65	90 96 96	84.1 (E)	ELISA	Plasma	11
Lawicki, S. 2017 [58]	Poland	Case-control	200 **Bc100** **B50** **H50** **48 (20-78)**	I/II/III: 77 IV: 23	VEGF+ CA 15-3	2 VEGF CA 15-3	78 (E) 60 64	90 95 95	84.7 (E)	ELISA	Plasma	11.5
Tjandra, JJ 1988 [60]	Australia	Case-control	161 **Bc109** **B31** **H21**	I: 32 II: 24 III/IV: 53	MSA + B2m	2 MSA B2m	88 (E) 88 39	90 95 90	88.5 (E)	Radioimmunoassay + ELISA	Serum	12
Ławicki, S 2013 [55]	Poland	Case-control	190 **Bc110** **B40** **H40** **44 (30-78**)	I: 25 II: 35 III: 25 IV: 25 (with metastases)	M-CSF + CA15-3	2 M-CSF CA 15-3	68 (E) 60 53	90 95 95	78.6 (E)	ELISA	Plasma	11
Ławicki, Sławomir 2013 [56]	Poland	Case-control	200 **Bc100** **B50** **H50** **51 (40–70)**	I/II/III: 75 IV: 25 (with metastases)	VEGF+ CA 15-3 M-CSF+ CA 15-3 VEGF + M-CSF VEGF+ M-CSF+ CA 15-3	2 2 2 3 VEGF M-CSF CA 15-3	63 (E) 57 (E) 60 (E) 68 (E) 44 53 36	86 86 86 78 92 94 92	76.4 (E) 73.9 (E) 75.2 (E) 73.8 (E)	ELISA	Plasma	11
Metwally, Fatehya M 2011 [61]	Egypt	Case-control	65 **Bc44** **H21** **36 (23–56)**	I: 21 II: 14 III: 9	CA 15.3 + IL-18	2 CA 15.3 IL-18	88.6 52.2 77.2	100 100 100	92.3	MEIA + ELISA + Colorimetric method	Serum	10.5

BC: breast cancer; B: benign; H: healthy; E: early stages (I, II, III); ELISA: the enzyme-linked immunosorbent assay; CMIA: luminescent microparticle immunoassay; MEIA: microparticle enzyme immunoassay. ^∗^It was made based on linear combination [35]. The scoring system based on the CASP checklist (specified for diagnostic studies) was applied for all studies.

**Table 2:** Relative effects and its 95% credible interval of all pairwise panels for sensitivity based on Bayesian network meta-analysis method.

	A	B	C	D	E	H	I	K	M
A	1								
B	**15.55 (1.8-159.59)**	1							
C	**9.88 (1.09-93.44)**	0.63 (0.24-1.56)	1						
D	7.87 (0.88-77.78)	0.51 (0.18-1.33)	0.8 (0.29-2.11)	1					
E	**14.87 (1.65-154.2)**	0.96 (0.35-2.56)	1.52 (0.59-4.03)	1.89 (0.69-5.49)	1				
H	4.81 (0.32-77.45)	0.31 (0.05-2.11)	0.49 (0.07-3.3)	0.61 (0.09-4.61)	0.32 (0.05-2.3)	1			
I	5.78 (0.38-96.46)	0.36 (0.05-2.74)	0.58 (0.09-4.18)	0.72 (0.1-5.68)	0.38 (0.05-2.82)	1.19 (0.09-15.06)	1		
K	2.53 (0.15-44.93)	0.16 (0.02-1.2)	0.25 (0.03-1.89)	0.32 (0.04-2.5)	0.17 (0.02-1.34)	0.52 (0.04-7.33)	0.44 (0.03-5.73)	1	
M	**11.61 (1.49-102.5)**	0.74 (0.34-1.58)	1.17 (0.57-2.44)	1.46 (0.61-3.61)	0.77 (0.33-1.82)	2.39 (0.4-14.24)	2.01 (0.33-12.07)	4.59 (0.7-30.51)	1

A: MMP-9/TIMP-1; B: M-CSF+CA15; C: VEGF + CA 15-3; D: VEGF + M-CSF + CA 15-3; E: VEGF+ M-CSF; H: CA 15.3 + IL-18; I: MSA + B2m; K: TF1+TF2+TF3; M: mammography.

**Table 3:** Ranking of different panels' sensitivity in early stages.

	Rank1	Rank2	Rank3	Rank4	Rank5	Rank6	Rank7	Rank8	Rank9
A	**0.674**	0.204	0.072	0.028	0.009	0.006	0.003	0.003	0.002
B	0.000	0.001	0.007	0.020	0.045	0.087	0.152	0.300	0.388
C	0.000	0.008	0.054	0.149	0.249	0.243	0.165	0.094	0.037
D	0.004	0.033	0.141	0.263	0.276	0.134	0.086	0.046	0.018
E	0.000	0.001	0.010	0.031	0.069	0.107	0.151	0.286	0.345
H	0.065	0.203	0.268	0.191	0.082	0.049	0.039	0.042	0.063
I	0.048	0.159	0.243	0.193	0.092	0.064	0.052	0.057	0.093
K	0.210	**0.390**	0.199	0.089	0.039	0.023	0.018	0.015	0.018
M	0.000	0.000	0.006	0.037	0.141	0.287	0.335	0.157	0.037

A: MMP-9/TIMP-1; B: M-CSF+CA15; C: VEGF + CA 15-3; D: VEGF + M-CSF + CA 15-3; E: VEGF+ M-CSF; H: CA 15.3 + IL-18; I: MSA + B2m; K: TF1+TF2+TF3; M: mammography.

**Table 4:** Relative effects and its 95% credible interval of all pairwise panels for specificity based on Bayesian network meta-analysis method.

	A	B	C	D	E	H	I	K	M
A	1								
B	**19.94 (1.63-293.1)**	1							
C	**30.24 (2.58-414.3)**	1.5 (0.44-5.04)	1						
D	**43.87 (3.68-669.7)**	2.2 (0.6-7.96)	1.46 (0.43-5.23)	1					
E	**25.69 (2.04-390.7)**	1.28 (0.36-4.48)	0.86 (0.25-2.94)	0.59 (0.16-2.16)	1				
H	0.52 (0.02-16.02)	**0.03 (0.01-0.37)**	**0.02 (0.0-0.24)**	**0.01 (0.0-0.17)**	**0.02 (0.0-0.29)**	1			
I	19.2 (0.62-737.3)	0.93 (0.05-18.96)	0.62 (0.04-12.4)	0.43 (0.02-9.48)	0.74 (0.04-15.66)	37.62 (0.94-1598.4)	1		
K	14.02 (0.41-518.4)	0.67 (0.04-12.82)	0.45 (0.03-9.09)	0.31 (0.02-6.33)	0.52 (0.03-10.81)	27.32 (0.75-954.7)	0.72 (0.01-36.91)	1	
M	2.92 (0.3-32.59)	**0.15 (0.05-0.4)**	**0.1 (0.03-0.26)**	**0.07 (0.02-0.21)**	**0.11 (0.04-0.36)**	5.71 (0.5-63.84)	0.16 (0.01-2.16)	0.22 (0.01-2.89)	1

A: MMP-9/TIMP-1; B: M-CSF+CA15; C: VEGF + CA 15-3; D: VEGF + M-CSF + CA 15-3; E: VEGF+ M-CSF; H: CA 15.3 + IL-18; I: MSA + B2m; K: TF1+TF2+TF3; M: mammography.

**Table 5:** Ranking of different panels' specificity in early stages.

	Rank1	Rank2	Rank3	Rank4	Rank5	Rank6	Rank7	Rank8	Rank9
A	0.327	**0.488**	0.117	0.048	0.012	0.005	0.002	0.001	0.001
B	0.000	0.000	0.004	0.139	0.310	0.281	0.162	0.082	0.022
C	0.000	0.000	0.001	0.032	0.109	0.215	0.270	0.256	0.117
D	0.000	0.000	0.001	0.009	0.035	0.079	0.164	0.302	0.410
E	0.000	0.000	0.002	0.071	0.178	0.247	0.243	0.178	0.082
H	**0.641**	0.273	0.055	0.021	0.006	0.002	0.001	0.000	0.001
I	0.008	0.026	0.056	0.237	0.181	0.089	0.088	0.099	0.216
K	0.014	0.042	0.086	0.308	0.160	0.083	0.071	0.082	0.153
M	0.010	0.170	0.677	0.134	0.009	0.000	0.000	0.000	0.000

A: MMP-9/TIMP-1; B: M-CSF+CA15; C: VEGF + CA 15-3; D: VEGF + M-CSF + CA 15-3; E: VEGF+ M-CSF; H: CA 15.3 + IL-18; I: MSA + B2m; K: TF1+TF2+TF3; M: mammography.

**Table 6:** Relative effects and the 95% credible interval of all pairwise panels for accuracy in early stages based on Bayesian network meta-analysis method.

	A	B	C	D	E	H	I	K	M
A	1								
B	**13.11 (3.63-64.32)**	1							
C	**12.8 (3.52-56.57)**	0.96 (0.59-1.55)	1						
D	**16.39 (4.52-78.16)**	1.23 (0.74-2.07)	1.28 (0.78-2.16)	1					
E	**12.53 (3.49-60.5)**	0.95 (0.57-1.59)	0.99 (0.59-1.65)	0.78 (0.45-1.29)	1				
H	2.03 (0.39-11.46)	**0.15 (0.04-0.49)**	**0.16 (0.05-0.52)**	**0.12 (0.04-0.4)**	**0.16 (0.05-0.52)**	1			
I	**6.41 (1.27-42.42)**	0.49 (0.15-1.51)	0.5 (0.16-1.59)	0.39 (0.12-1.27)	0.51 (0.16-1.6)	3.22 (0.7-15.51)	1		
K	3.78 (0.7-22.83)	**0.28 (0.08-0.95)**	**0.29 (0.09-0.96)**	**0.23 (0.07-0.78)**	0.3 (0.08-1.01)	1.83 (0.39-9.56)	0.58 (0.12-2.8)	1	
M	**6.87 (2.07-31.35)**	**0.53 (0.35-0.78)**	**0.55 (0.37-0.81)**	**0.43 (0.27-0.68)**	**0.55 (0.35-0.88)**	**3.44 (1.15-11.07)**	1.09 (0.37-3.22)	1.88 (0.6-5.87)	1

A: MMP-9/TIMP-1; B: M-CSF+CA15; C: VEGF + CA 15-3; D: VEGF + M-CSF + CA 15-3; E: VEGF+ M-CSF; H: CA 15.3 + IL-18; I: MSA + B2m; K: TF1+TF2+TF3; M: mammography.

**Table 7:** Ranking of different panels' accuracy in early stages.

	Rank1	Rank2	Rank3	Rank4	Rank5	Rank6	Rank7	Rank8	Rank9
A	**0.768**	0.191	0.035	0.005	0.000	0.000	0.000	0.000	0.000
B	0.000	0.000	0.000	0.002	0.048	0.243	0.279	0.299	0.129
C	0.000	0.000	0.000	0.004	0.059	0.316	0.293	0.232	0.096
D	0.000	0.000	0.000	0.000	0.008	0.045	0.108	0.214	0.624
E	0.000	0.000	0.001	0.009	0.078	0.321	0.275	0.214	0.103
H	0.189	**0.567**	0.203	0.032	0.006	0.001	0.001	0.000	0.001
I	0.005	0.043	0.200	0.337	0.247	0.057	0.035	0.034	0.042
K	0.038	0.198	0.492	0.163	0.076	0.014	0.008	0.007	0.005
M	0.000	0.001	0.069	0.448	0.478	0.004	0.000	0.000	0.000

A: MMP-9/TIMP-1; B: M-CSF+CA15; C: VEGF + CA 15-3; D: VEGF + M-CSF + CA 15-3; E: VEGF+ M-CSF; H: CA 15.3 + IL-18; I: MSA + B2m; K: TF1+TF2+TF3; M: mammography.

## Data Availability

The data used to support the findings of this study are available from the corresponding author upon request.

## Supplementary Materials

Supplementary Materials Supplementary Material 1 Appendices: A includes risk of bias studies, traditional meta-analysis of all panels in early stages, and node-splitting analysis of inconsistency for sensitivity, specificity, and accuracy. B contains the search strategy for each database. C includes the non-English articles and inaccessible articles. D has the identified fifty-four studies relevant to our research question. E contains the list of multistudy panels and selected single-study panels. F includes the preanalytical procedures.

## References

[1] Sabatino S. A., Lawrence B., Elder R. (2012). Effectiveness of interventions to increase screening for breast, cervical, and colorectal cancers: nine updated systematic reviews for the guide to community preventive services. *American Journal of Preventive Medicine*.

[2] Banegas M. P., Bird Y., Moraros J., King S., Prapsiri S., Thompson B. (2012). Breast cancer knowledge, attitudes, and early detection practices in United States-Mexico border Latinas. *Journal of Women's Health*.

[3] Motamedi M. H. K., Nafissi N., Saghafinia M., Akbari M. E. (2012). A survey of breast cancer knowledge and attitude in Iranian women. *Journal of Cancer Research and Therapeutics*.

[4] Okobia M. N., Bunker C. H., Okonofua F. E., Osime U. (2006). Knowledge, attitude and practice of Nigerian women towards breast cancer: a cross-sectional study. *World Journal of Surgical Oncology*.

[5] Saika K., Sobue T. (2009). Epidemiology of breast cancer in Japan and the US. *JMAJ*.

[6] Baquet C. R., Commiskey P. (2000). Socioeconomic factors and breast carcinoma in multicultural women. *Cancer*.

[7] Berry D. A., Cronin K. A., Plevritis S. K. (2005). Effect of screening and adjuvant therapy on mortality from breast cancer. *The New England Journal of Medicine*.

[8] Sadjadian A., Kaviani A., Yunesian M., Montazeri A. (2004). Patient satisfaction: a descriptive study of a breast care clinic in Iran. *European Journal of Cancer Care*.

[9] Cardoso F., Kyriakides S., Ohno S. (2019). Early breast cancer: ESMO Clinical Practice Guidelines for diagnosis, treatment and follow-up^†^. *Annals of Oncology*.

[10] Hortobagyi G. N., de la Garza Salazar J., Pritchard K. (2005). The global breast cancer burden: variations in epidemiology and survival. *Clinical Breast Cancer*.

[11] Lertkhachonsuk A.-a., Yip C. H., Khuhaprema T. (2013). Cancer prevention in Asia: resource-stratified guidelines from the Asian Oncology Summit 2013. *The Lancet Oncology*.

[12] Moodi M., Rezaeian M., Mostafavi F., Sharifirad G. R. (2012). Determinants of mammography screening behavior in Iranian women: a population-based study. *Journal of Research in Medical Sciences : The Official Journal of Isfahan University of Medical Sciences*.

[13] Grunfeld E., Ramirez A., Hunter M., Richards M. A. (2002). Women’s knowledge and beliefs regarding breast cancer. *British Journal of Cancer*.

[14] Hadi N., Sadeghi Hassanabadi A., Talei A. R., Arasteh M. M., Kazerooni T. (2002). Assessment of a breast cancer screening programme in Shiraz, Islamic Republic of Iran. *EMHJ-Eastern Mediterranean Health Journal*.

[15] Haji-Mahmoodi M., Montazeri A., Jarvandi S., Ebrahimi M., Haghighat S., Harirchi I. (2002). Breast self-examination: knowledge, attitudes, and practices among female health care workers in Tehran, Iran. *The Breast Journal*.

[16] Heidari Z., Mahmoudzadeh-Sagheb H., Sakhavar N. (2008). Breast cancer screening knowledge and practice among women in southeast of Iran. *Acta Medica Iranica*.

[17] Hoskins C. N., Haber J. (2000). Adjusting to breast cancer. *AJN The American Journal of Nursing*.

[18] Ibrahim N. A., Odusanya O. O. (2009). Knowledge of risk factors, beliefs and practices of female healthcare professionals towards breast cancer in a tertiary institution in Lagos, Nigeria. *BMC Cancer*.

[19] McMenamin M., Barry H., Lennon A.-M. (2005). A survey of breast cancer awareness and knowledge in a Western population: lots of light but little illumination. *European Journal of Cancer*.

[20] Najah K. (2010). *Breast cancer risk-factors and breast self examination practice among Jordanian women*.

[21] Novak E. (2007). *Berek & Novak's Gynecology*.

[22] Preston D. L., Mattsson A., Holmberg E., Shore R., Hildreth N. G., Boice J. D. (2002). Radiation effects on breast cancer risk: a pooled analysis of eight cohorts. *Radiation Research*.

[23] Smeltzer S. C., Bare B. G., Hinkle J. L., Cheever K. H., Townsend M. C., Gould B. (2008). *Brunner and Suddarth’s Textbook of Medicalsurgical Nursing*.

[24] Travis L. B., Hill D. A., Dores G. M. (2003). Breast cancer following radiotherapy and chemotherapy among young women with Hodgkin disease. *Journal of the American Medical Association*.

[25] Al Junaibi R. M., Khan S. A. (2011). Knowledge and awareness of breast cancer among university female students in Muscat, Sultanate of Oman-a pilot study. *Journal of Applied Pharmaceutical Science*.

[26] Pinnoti J. A., Barros A. C., Hegg R., Zeferino L. C. (1995). Breast cancer program in developing countries. *Breast Diseases*.

[27] Berg W. A., Mrose H. E., Ioffe O. B. (2001). Atypical lobular hyperplasia or lobular carcinoma in situ at core-needle breast biopsy. *Radiology*.

[28] Schrager S., Marko K. (2013). Mammography at age 40? A risk-based strategy. *The Journal of Family Practice*.

[29] Li J., Guan X., Fan Z. (2020). Non-invasive biomarkers for early detection of breast cancer. *Cancers*.

[30] Achimaş-Cadariu P., Irimie A., Achimaş-Cadariu L., Neagoe I., Buiga R. (2009). Could serologic and ultrasonographic indexes be useful for therapeutic decisions in patients with ovarian cancer?. *Chirurgia*.

[31] Cristofanilli M., Budd G. T., Ellis M. J. (2004). Circulating tumor cells, disease progression, and survival in metastatic breast cancer. *The New England Journal of Medicine*.

[32] Hayes D. F., Cristofanilli M., Budd G. T. (2006). Circulating tumor cells at each follow-up time point during therapy of metastatic breast cancer patients predict progression-free and overall survival. *Clinical Cancer Research*.

[33] Zhang L., Riethdorf S., Wu G. (2012). Meta-analysis of the prognostic value of circulating tumor cells in breast cancer. *Clinical Cancer Research*.

[34] Moher D., Liberati A., Tetzlaff J., Altman D. G., The PRISMA Group (2009). Preferred reporting items for systematic reviews and meta-analyses: the PRISMA statement. *PLoS Medicine*.

[35] Pepe M. S., Thompson M. L. (2000). Combining diagnostic test results to increase accuracy. *Biostatistics*.

[36] Swellam M., Soliman H. A., Abdelmaksoud M. D., Nageeb A. M., el Arab L. R. E., Boshnak H. (2014). Clinical implications of proteolytic activity imbalance in breast cancer diagnosis. *Cancer Biomarkers*.

[37] Ontario H. Q. (2007). Screening mammography for women aged 40 to 49 years at average risk for breast cancer: an evidence-based analysis. *Ontario health technology assessment series*.

[38] Sinclair N., Littenberg B., Geller B., Muss H. (2011). Accuracy of screening mammography in older women. *American Journal of Roentgenology*.

[39] Yankaskas B. C., Haneuse S., Kapp J. M. (2010). Performance of first mammography examination in women younger than 40 years. *JNCI: Journal of the National Cancer Institute*.

[40] Higgins J. P., Thompson S. G. (2002). Quantifying heterogeneity in a meta-analysis. *Statistics in Medicine*.

[41] Huedo-Medina T. B., Sánchez-Meca J., Marín-Martínez F., Botella J. (2006). Assessing heterogeneity in meta-analysis: Q statistic or I^2^ index?. *Psychological Methods*.

[42] Hu D., O'Connor A. M., Wang C., Sargeant J. M., Winder C. B. (2020). How to conduct a Bayesian network meta-analysis. *Science*.

[43] Tu Y. K., Needleman I., Chambrone L., Lu H. K., Faggion C. M. (2012). A Bayesian network meta-analysis on comparisons of enamel matrix derivatives, guided tissue regeneration and their combination therapies. *Journal of Clinical Periodontology*.

[44] Jansen J. P., Fleurence R., Devine B. (2011). Interpreting indirect treatment comparisons and network meta-analysis for health-care decision making: report of the ISPOR Task Force on Indirect Treatment Comparisons Good Research Practices: part 1. *Value in Health*.

[45] Salanti G., del Giovane C., Chaimani A., Caldwell D. M., Higgins J. P. T. (2014). Evaluating the quality of evidence from a network meta-analysis. *PLoS One*.

[46] Dias S., Welton N. J., Caldwell D., Ades A. E. (2010). Checking consistency in mixed treatment comparison meta-analysis. *Statistics in Medicine*.

[47] Yu-Kang T. (2016). Node-splitting generalized linear mixed models for evaluation of inconsistency in network meta-analysis. *Value in Health*.

[48] Smith A. E., Kulasekararaj A. G., Jiang J. (2015). _CSNK1A1_ mutations and isolated del(5q) abnormality in myelodysplastic syndrome: a retrospective mutational analysis. *The Lancet Haematology*.

[49] Tonin F. S., Rotta I., Mendes A. M., Pontarolo R. (2017). Network meta-analysis: a technique to gather evidence from direct and indirect comparisons. *Pharmacy Practice*.

[50] Veroniki A. A., Straus S. E., Fyraridis A., Tricco A. C. (2016). The rank-heat plot is a novel way to present the results from a network meta- analysis including multiple outcomes. *Journal of Clinical Epidemiology*.

[51] Rücker G., Schwarzer G., Krahn U. (2015). *Package ‘netmeta’*.

[52] Schwarzer G., Schwarzer M. G. (2012). *Package ‘meta’*.

[53] van Valkenhoef G., Kuiper J., van Valkenhoef M. G. (2016). *Package ‘gemtc’*.

[54] Ishibashi Y., Ohtsu H., Ikemura M. (2017). Serum TFF1 and TFF3 but not TFF2 are higher in women with breast cancer than in women without breast cancer. *Scientific Reports*.

[55] Ławicki S., Będkowska G., Wojtukiewicz M., Szmitkowski M. (2013). Hematopoietic cytokines as tumor markers in breast malignancies. A multivariate analysis with ROC curve in breast cancer patients. *Advances in Medical Sciences*.

[56] Ławicki S., Będkowska G. E., Szmitkowski M. (2013). VEGF, M-CSF and CA 15-3 as a new tumor marker panel in breast malignancies: a multivariate analysis with ROC curve. *Growth Factors*.

[57] Ławicki S., Zajkowska M., Głażewska E. K., Będkowska G. E., Szmitkowski M. (2016). Plasma levels and diagnostic utility of VEGF, MMP-9, and TIMP-1 in the diagnosis of patients with breast cancer. *OncoTargets and Therapy*.

[58] Ławicki S., Zajkowska M., Głażewska E. K., Będkowska G. E., Szmitkowski M. (2017). Plasma levels and diagnostic utility of VEGF, MMP-2 and TIMP-2 in the diagnostics of breast cancer patients. *Biomarkers*.

[59] Zajkowska M., Głażewska E. K., Będkowska G. E., Chorąży P., Szmitkowski M., Ławicki S. (2016). Diagnostic power of vascular endothelial growth factor and macrophage colony- stimulating factor in breast cancer patients based on ROC analysis. *Mediators of Inflammation*.

[60] Tjandra J., McLaughlin P., Russell I., Collins J. P., McKenzie I. F. C. (1988). Comparison of mammary serum antigen (MSA) with *β*_2_-microglobulin (*β*_2_M) and carcinoembryonic antigen (CEA) assays in patients with breast cancer. *European Journal of Cancer & Clinical Oncology*.

[61] Metwally F. M., el-mezayen H. A., Ahmed H. H. (2011). Significance of vascular endothelial growth factor, interleukin-18 and nitric oxide in patients with breast cancer: correlation with carbohydrate antigen 15.3. *Medical Oncology*.

[62] Stellman S. D., Gordis L. (2010). *Book Review: Epidemiology*.

