# Supplementary material for: Diagnostic Value of Nonacid Nucleic Blood Tumor Marker Panels in Early Diagnosing Breast Cancer: A Systematic Review and Network Meta-Analysis
Source: Dis Markers. 2022 Feb 3;2022:4119345. doi: 10.1155/2022/4119345 (PMC8866026; doi:10.1155/2022/4119345)
Supplement: Supplementary Materials — Supplementary Material 1 Appendices: A includes risk of bias studies, traditional meta-analysis of all panels in early stages, and node-splitting analysis of inconsistency for sensitivity, specificity, and accuracy. B contains the search strategy for each database. C includes the non-English articles and inaccessible articles. D has the identified fifty-four studies relevant to our research question. E contains the list of multistudy panels and selected single-study panels. F includes the preanalytical procedures. [file 4119345.f1.docx]

**Title**: Diagnostic value of non-acid nucleic blood tumor markers panels in early diagnosing breast cancer: A systematic review and network meta-analysis

**Journal name:** disease markers, our manuscript is not under review or published elsewhere.

**Author names:**

1. Vahid Raja, Clinical Laboratory Sciences, Amin Hospital, Isfahan university of Medical sciences, Isfahan, Iran. E-mail:mahantajhizs@gmail.com- [amin@mui.ac.ir-](mailto:amin@mui.ac.ir-) ORCID number: 0000-0003-4655-6947

2. Ziba Farajzadegan, professor of community & preventive medicine, Isfahan university of Medical sciences. Isfahan, Iran.

E-mail:farajzadegan@med.mui.ac.ir- Tel: 091-3291266 - ORCID number: 0000-0001-8548-28433. Marjan Mansourian, professor in Biostatistics, Department of Biostatistics and Epidemiology, School of public Health, Isfahan university of Medical sciences, Isfahan, Iran. E-mail:J_mansourian@hlth.mui.ac.ir

4. Khojaste Ghasemi, Department of Biostatistics and Epidemiology, School of public Health, Isfahan university of Medical sciences, Isfahan, Iran. Email:khojastehghasemi1372@gmail.com

5. Mohammad Sadegh Aboutalebi, Faculyt of nursing and midwifery, Isfahan university of Medical sciences, Isfahan, Iran. Email:Aboutalebi@nm.mu.ac.ir

6. Rasool Nouri, Assistant professor in Department of medical library and information sciences, Isfahan university of Medical sciences, Isfahan, Iran. Email:nouri@mng.mui.ac.ir

7. Fariborz Mokarian, Assistant Professor of Hematology & Oncology Department of Internal Medicine, Isfahan university of Medical sciences, Isfahan, Iran. Email:Frajabi41@gmail.com

Author contributions

Vahid Raja had the idea for the article. The literature search was performed by Vahid Raja, Mohammad Sadegh Aboutalebi and Rasool Nouri. The data analysis was performed by Marjan Mansourian and Khojaste Ghasemi. The article was drafted by Vahid Raja and Ziba Farajzadegan. Critically revised the article was performed by Vahid Raja, Ziba Farajzadegan and Fariborz Mokarian.

**Appendix A**: Includes of risk of bias studies, traditional meta-analysis of all panels in early stages, nod-splitting analysis of inconsistency for sensitivity, specificity and accuracy.

***Table 1:* Risk of bias.**

| ­­­­**Risk of bias** | **Score** | **Study design** | **Title** | **First author and year** |
| --- | --- | --- | --- | --- |
| Blinding test was not mentioned. Sensitivity and specificity were not high. | 11 | Case-control | Diagnostic Power of Vascular Endothelial Growth Factor and Macrophage Colony-Stimulating Factor in Breast Cancer Patients Based on ROC Analysis | S Zajkowska, M..  2016 |
| Blinding test was not mentioned Standard test was not mentioned. | 11 | Cohort | Serum TFF1 and TFF3 but not TFF2 are higher in women with breast cancer than in women without breast cancer | Ikemura, M.  2017 |
| Blinding test was not mentioned. Author was not sure about efficiency of the test. | 11 | Case-control | Clinical implications of proteolytic activity imbalance in breast cancer diagnosis | Swellam, M.  2014 |
| Utilized standard test was not exactly mentioned. Blinding test was not mentioned. | 11 | Case-control | Plasma levels and diagnostic utility of VEGF, MMP-9, and TIMP-1 in the diagnosis of patients with breast cancer | Ławicki, Sławomir  2016 |
| Blinding test was not mentioned. | 11.5 | Case-control | Plasma levels and diagnostic utility of VEGF, MMP-2 and TIMP-2 in the diagnostics of breast cancer patients | Lawicki, S.  2017 |

|  | 12 | Case-control | Comparison of mammary serum antigen (MSA) with β2-microglobulin (β2M) and carcinoembryonic antigen (CEA) assays in patients with breast cancer | Tjandra, JJ  1988 |
| --- | --- | --- | --- | --- |
| Blinding test was not mentioned. Sensitivity and specificity were low. | 11 | Case-control | Hematopoietic cytokines as tumor markers in breast malignancies. A multivariate analysis with ROC curve in breast cancer patients | Ławicki, S  2013 |
| Blinding test was not mentioned. Sensitivity and specificity were comparatively low. | 11 | Case-control | VEGF, M-CSF and CA 15-3 as a new tumor marker panel in breast malignancies: a multivariate analysis with ROC curve | Ławicki, Sławomir  2013 |
| There was probability of verification bias. Blinding test was not mentioned.  SAMPLE SIZE: 65 | 10.5 | Case-control | Significance of vascular endothelial growth factor, interleukin-18 and nitric oxide in patients with breast cancer: correlation with carbohydrate antigen 15.3 | Metwally, Fatehya M  2011 |

**Table 2: Traditional meta-analysis of all panels in early stages.**

| Study | | Comparisons | | OR (95%CL) | | P-value | | I^2^ | | Τ^2^ |  |
| --- | --- | --- | --- | --- | --- | --- | --- | --- | --- | --- | --- |
| Sensitivity | |  | |  | |  | |  | |  |  |
| 1 study | | A - M | | 24.20 (3.24 - 53.64) | | 0.005 | | - | | - |  |
| 3 studies | | B - M | | 0.76 (0.30 - 1.92) | | 0.574 | | 82.5% | | 0.54 |  |
| 4 studies | | C - M | | 1.07 (0.47 - 2.4) | | 0.890 | | 84.9% | | 0.74 |  |
| 2 studies | | D - M | | 1.62 (0.19 - 13.9) | | 0.656 | | 92.2% | | 2.21 |  |
| 2 studies | | E - M | | 0.74 (0.19 - 2.6) | | 0.673 | | 87.2% | | 0.81 |  |
| 1 study | | H - M | | 2.31 (1.08-4.94) | | 0.029 | | - | | - |  |
| 1 study | | I - M | | 1.94 (0.9 – 4.2) | | 0.090 | | - | | - |  |
| 1 study | | K - M | | 2.39 (1.06 – 5.38) | | 0.035 | | - | | - |  |
| 2 studies | | B - C | | 0.69 (0.28 - 1.72) | | 0.437 | | 57.3% | | 0.25 |  |
| 2 studies | | B - D | | 0.56 (0.33 - 0.93) | | 0.026 | | 10.0% | | 0.001 |  |
| 2 studies | | B - E | | 1.09 (0.47 - 2.63) | | 0.908 | | 8.9% | | 0.001 |  |
| 2 studies | | C - D | | 0.83 (0.39 - 1.14) | | 0.144 | | 8.7% | | 0.001 |  |
| 2 studies | | C - E | | 1.38 (0.84 – 3.69) | | 0.290 | | 54.4% | | 0.649 |  |
| 2 studies | | D - E | | 1.72 (0.99 – 3.17) | | 0.052 | | 20.3% | | 0.014 |  |
| Specificity | |  | |  | |  | |  | |  |  |
| 1 study | | A - M | | 2.8 (0.97 –8.29) | | 0.056 | | - | | - |  |
| 3 studies | | B - M | | 0.24 (0.01 – 3.36) | | 0.289 | | 92.6% | | 5.01 |  |
| 4 studies | | C - M | | 0.12 (0.05 – 0.23) | | <0.001 | | 0.0% | | 0.001 |  |
| 2 studies | | D - M | | 0.04 (0.01 – 0.12) | | <0.001 | | 0.0% | | 0.001 |  |
| 2 studies | | E - M | | 0.09 (0.03 – 0.25) | | <0.001 | | 6.3% | | 0.040 |  |
| 1 study | | H - M | | 23.01 (1.8 – 112.4) | | 0.017 | | - | | - |  |
| 1 study | | I - M | | 0.18 (0.03-0.86) | | 0.031 | | - | | - |  |
| 1 study | | K - M | | 0.27 (0.05 – 1.33) | | 0.109 | | - | | - |  |
| 2 studies | | B - C | | 1.05 ( 0.66 – 1.69) | | 0.809 | | 0.0% | | 0.001 |  |
| 2 studies | | B - D | | 1.60 (1.02- 2.52) | | 0.040 | | 0.0% | | 0.001 |  |
| 2 studies | | B - E | | 0.75 (0.46 -1.23) | | 0.266 | | 0.0% | | 0.001 |  |
| 2 studies | | C - D | | 1.51 (0.96 – 2.38) | | 0.072 | | 0.0% | | 0.001 |  |
| 2 studies | | C - E | | 0.71 (0.43 – 1.16) | | 0.178 | | 7.0% | | 0.099 |  |
| 2 studies | | D - E | | 0.41 (0.22 – 0.76) | | 0.040 | | 0.0% | | 0.001 |  |
| Accuracy |  | |  | |  | |  | |  | | |
| 1 study | A - M | | 6.00 (1.96 - 18.27) | | 0.001 | | - | | - | | |
| 3 studies | B - M | | 0.58 (0.29 - 1.16) | | 0.125 | | 62.9% | | 0.23 | | |
| 4 studies | C - M | | 1.11 (0.78 - 1.57) | | 0.540 | | 0.0% | | 0.001 | | |
| 2 studies | D - M | | 0.38 (0.22 - 0.65) | | 0.005 | | 0.0% | | 0.001 | | |
| 2 studies | E - M | | 0.48 (0.28 - 0.83) | | 0.008 | | 0.0% | | 0.001 | | |
| 1 study | H - M | | 3.91 (1.5-10.22) | | 0.005 | | - | | - | | |
| 1 study | I - M | | 1.10 (0.46 – 2.63) | | 0.824 | | - | | - | | |
| 1 study | K - M | | 1.81 (0.68 – 4.81) | | 0.233 | | - | | - | | |
| 2 studies | B - C | | 0.92 (0.58 - 1.45) | | 0.724 | | 0.0% | | 0.001 | | |
| 2 studies | B - D | | 1.08 (0.68 - 1.70) | | 0.730 | | 0.0% | | 0.001 | | |
| 2 studies | B - E | | 0.84 (0.52 - 1.34) | | 0.476 | | 0.0% | | 0.001 | | |
| 2 studies | C - D | | 1.21 (0.76 - 1.91) | | 0.144 | | 3.1% | | 0.001 | | |
| 2 studies | C - E | | 0.94 (0.58 – 1.51) | | 0.809 | | 0.0% | | 0.001 | | |
| 2 studies | D - E | | 0.77 (0.49 – 1.23) | | 0.292 | | 6.5% | | 0.001 | | |

A= MMP-9/TIMP-1, B= M-CSF+CA15, C= VEGF + CA 15-3, D= VEGF + M-CSF + CA 15-3, E= VEGF+ M-CSF, H= CA 15.3 + IL-18, I= MSA + B2m, K= TF1+TF2+TF3, M=Mammography

**Table 3: Nod-splitting analysis of inconsistency for sensitivity in early stages**

| Comparisons | | Mean difference (95%CrI) | P-value |
| --- | --- | --- | --- |
| B vs. C | Direct | 0.57 (-0.96, 2.2) | 0.835975 |
|  | Indirect | 0.36 (-1.3, 2.0) |  |
|  | Network | 0.45 (-0.49, 1.4) |  |
| B vs. D | Direct | 0.71 (-0.87, 2.3) | 0.992575 |
|  | Indirect | 0.72 (-1.2, 2.8) |  |
|  | Network | 0.69 (-0.33, 1.7) |  |
| B vs. E | Direct | 0.017 (-1.5, 1.5) | 0.959675 |
|  | Indirect | 0.072 (-1.9, 2.0) |  |
|  | Network | 0.033 (-0.98, 1.0) |  |
| B vs. M | Direct | 0.26 (-0.84, 1.4) | 0.926425 |
|  | Indirect | 0.34 (-1.0, 1.6) |  |
|  | Network | 0.30 (-0.52, 1.1) |  |
| C vs. D | Direct | 0.13 (-1.4, 1.7) | 0.839850 |
|  | Indirect | 0.36 (-1.5, 2.2) |  |
|  | Network | 0.23 (-0.79, 1.3) |  |
| C vs. E | Direct | -0.55 (-2.1, 1.0) | 0.815025 |
|  | Indirect | -0.29 (-2.2, 1.6) |  |
|  | Network | -0.42 (-1.5, 0.56) |  |
| C vs. M | Direct | -0.090 (-1.1, 0.86) | 0.772925 |
|  | Indirect | -0.31 (-1.7, 1.0) |  |
|  | Network | -0.16 (-0.93, 0.57) |  |
| D vs. E | Direct | -0.68 (-2.3, 0.88) | 0.978750 |
|  | Indirect | -0.65 (-2.9, 1.5) |  |
|  | Network | -0.66 (-1.7, 0.39) |  |
| D vs. M | Direct | -0.44 (-1.9, 0.91) | 0.937550 |
|  | Indirect | -0.37 (-1.7, 0.95) |  |
|  | Network | -0.39 (-1.3, 0.49) |  |
| E vs. M | Direct | 0.21 (-1.2, 1.6) | 0.896300 |
|  | Indirect | 0.32 (-0.98, 1.6) |  |
|  | Network | 0.27 (-0.63, 1.2) |  |

A= MMP-9/TIMP-1, B= M-CSF+CA15, C= VEGF + CA 15-3, D= VEGF + M-CSF + CA 15-3, E= VEGF+ M-CSF, H= CA 15.3 + IL-18, I= MSA + B2m, K= TF1+TF2+TF3, M=Mammography

**Table 4: Nod-splitting analysis of inconsistency for specificity in early stages**

| Comparisons | | Mean difference  (95%CI) | P-value |
| --- | --- | --- | --- |
| B vs. C | Direct | -0.048 (-1.9, 1.8) | 0.467100 |
|  | Indirect | -1.0 (-3.1, 1.2) |  |
|  | Network | -0.41 (-1.7, 0.90) |  |
| B vs. D | Direct | -0.18 (-2.0, 1.7) | 0.243150 |
|  | Indirect | -1.9 (-4.5, 0.70) |  |
|  | Network | -0.76 (-2.1, 0.62) |  |
| B vs. E | Direct | 0.23 (-1.6, 2.1) | 0.332825 |
|  | Indirect | -1.2 (-3.8, 1.4) |  |
|  | Network | -0.26 (-1.7, 1.1) |  |
| B vs. M | Direct | 1.3 (-0.011, 2.9) | 0.209550 |
|  | Indirect | 2.7 (1.1, 4.3) |  |
|  | Network | 1.9 (0.87, 3.1) |  |
| C vs. D | Direct | -0.13 (-2.1, 1.8) | 0.587550 |
|  | Indirect | -0.95 (-3.6, 1.7) |  |
|  | Network | -0.34 (-1.7, 1.0) |  |
| C vs. E | Direct | 0.28 (-1.7, 2.3) | 0.743350 |
|  | Indirect | -0.23 (-3.0, 2.5) |  |
|  | Network | 0.15 (-1.2, 1.5) |  |
| C vs. M | Direct | 2.4 (0.95, 3.8) | 0.977400 |
|  | Indirect | 2.3 (0.58, 4.2) |  |
|  | Network | 2.4 (1.3, 3.5) |  |
| D vs. E | Direct | 0.42 (-1.5, 2.3) | 0.845900 |
|  | Indirect | 0.73 (-2.3, 3.8) |  |
|  | Network | 0.50 (-0.94, 1.9) |  |
| D vs. M | Direct | 3.3 (1.4, 5.4) | 0.395975 |
|  | Indirect | 2.3 (0.63, 4.0) |  |
|  | Network | 2.7 (1.5, 4.9) |  |
| E vs. M | Direct | 2.6 (0.64, 4.7) | 0.602475 |
|  | Indirect | 1.9 (0.28, 3.7) |  |
|  | Network | 2.2 (0.99, 3.5) |  |

A= MMP-9/TIMP-1, B= M-CSF+CA15, C= VEGF + CA 15-3, D= VEGF + M-CSF + CA 15-3, E= VEGF+ M-CSF, H= CA 15.3 + IL-18, I= MSA + B2m, K= TF1+TF2+TF3, M=Mammography

**Table 5: Nod-splitting analysis of inconsistency for accuracy in early stages**

| Comparisons | | Mean difference  (95%CI) | P-value |
| --- | --- | --- | --- |
| B vs. C | Direct | 0.078 (-0.48, 0.63) | 0.824200 |
|  | Indirect | -0.0042 (-0.66, 0.65) |  |
|  | Network | 0.040 (-0.34, 0.44) |  |
| B vs. D | Direct | -0.075 (-0.63, 0.47) | 0.448275 |
|  | Indirect | -0.43 (-1.2, 0.34) |  |
|  | Network | -0.19 (-0.59, 0.23) |  |
| B vs. E | Direct | 0.16 (-0.39, 0.72) | 0.499200 |
|  | Indirect | -0.15 (-0.95, 0.64) |  |
|  | Network | 0.065 (-0.35, 0.48) |  |
| B vs. M | Direct | 0.54 (0.082, 1.0) | 0.477100 |
|  | Indirect | 0.79 (0.26, 1.3) |  |
|  | Network | 0.64 (0.31, 1.0) |  |
| C vs. D | Direct | -0.16 (-0.74, 0.39) | 0.604800 |
|  | Indirect | -0.40 (-1.2, 0.35) |  |
|  | Network | -0.23 (-0.66, 0.19) |  |
| C vs. E | Direct | 0.094 (-0.47, 0.66) | 0.601050 |
|  | Indirect | -0.14 (-0.90, 0.64) |  |
|  | Network | 0.021 (-0.40, 0.43) |  |
| C vs. M | Direct | 0.56 (0.13, 1.0) | 0.803300 |
|  | Indirect | 0.65 (0.11, 1.2) |  |
|  | Network | 0.60 (0.27, 0.93) |  |
| D vs. E | Direct | 0.24 (-0.30, 0.81) | 0.994275 |
|  | Indirect | 0.25 (-0.62, 1.1) |  |
|  | Network | 0.25 (-0.19, 0.68) |  |
| D vs. M | Direct | 0.95 (0.38, 1.5) | 0.619525 |
|  | Indirect | 0.75 (0.24, 1.3) |  |
|  | Network | 0.83 (0.45, 1.2) |  |
| E vs. M | Direct | 0.70 (0.11, 1.3) | 0.595900 |
|  | Indirect | 0.50 (-0.030, 1.0) |  |
|  | Network | 0.58 (0.19, 0.97) |  |

A= MMP-9/TIMP-1, B= M-CSF+CA15, C= VEGF + CA 15-3, D= VEGF + M-CSF + CA 15-3, E= VEGF+ M-CSF, H= CA 15.3 + IL-18, I= MSA + B2m, K= TF1+TF2+TF3, M=Mammography

**Appendix B**: The search strategy for each data base.

Web of Science: 715

# 1 136647

TOPIC: ("breast cancer mucin" OR "cancer antigen27.29" OR "cancer antigen15.3" OR "cancer antigen125" OR "cancer antigen549" OR "cancer associated serum antigen" OR "catapsin d" OR "carcinoma embryonic antigen" OR "creatine kinase-brain" OR "carboxypeptidase n") OR TOPIC: ("collapsin response mediator proteins" OR "colony stimulating factor1" OR "cytokeratin fragment" OR "Galectin3" OR "human epidermal growth factor receptor-2" OR "Human mammaglobin") OR TOPIC: ("mannose receptor" OR "mucin- like carcinoma" OR "Matrix metalloproteinase-9" OR "Mammary serum antigen" OR "Nicotinamide phosphoribosyl transferase" OR "p53 protein") OR TOPIC: ("phosphohexose isomerase" OR "small breast epithelial mucin" OR survivin OR "Tumor-associated glycoprotein72" OR "Tissue Inhibitor of Metalloproteinases-1" OR "Thymidine kinase" OR "Tissue polypeptide antigen" OR "Tissue polypeptide-specific antigen") OR TOPIC: ("Urokinase plasminogen activator" OR "plasminogen activator inhibitor-1" OR "Vascular Endothelial Growth Factor") OR TOPIC: ("E_selectin:endothelial selectin" OR "P-selectin:platelet selectin" OR "intracellular adhesion molecule 1")

Indexes=SCI-EXPANDED, SSCI, A&HCI, ESCI Timespan=All years Edit

2# 469946

TOPIC: ("Breast Neoplasms" OR "breast cancer")")

Indexes=SCI-EXPANDED, SSCI, A&HCI, ESCI Timespan=All years

3# 927123

TS=("Early Detection" OR "Early Diagnosis" OR screening))

Indexes=SCI-EXPANDED, SSCI, A&HCI, ESCI Timespan=All years

4# 715

#3 AND #2 AND #1

Indexes=SCI-EXPANDED, SSCI, A&HCI, ESCI Timespan=All years

Med line: 432

#1 117606

Add

Search ("breast cancer mucin"[Title/Abstract] OR "cancer antigen27.29"[Title/Abstract] OR cancer antigen15.3[Title/Abstract] OR "cancer antigen125"[Title/Abstract] OR "cancer antigen549"[Title/Abstract] OR "cancer associated serum antigen"[Title/Abstract] OR "catapsin d"[Title/Abstract] OR "carcinoma embryonic antigen"[Title/Abstract] OR "creatine kinase-brain"[Title/Abstract] OR "carboxypeptidase n"[Title/Abstract] OR "collapsin response mediator proteins"[Title/Abstract] OR "colony stimulating factor1"[Title/Abstract] OR "cytokeratin fragment"[Title/Abstract] OR "Galectin.3"[Title/Abstract] OR "human epidermal growth factor receptor-2"[Title/Abstract] OR "Human mammaglobin"[Title/Abstract] OR "mannose receptor"[Title/Abstract] OR "mucin- like carcinoma"[Title/Abstract] OR "Matrix metalloproteinase-9"[Title/Abstract] OR "Mammary serum antigen"[Title/Abstract] OR "Nicotinamide phosphoribosyl transferase"[Title/Abstract] OR "p53 protein"[Title/Abstract] OR "phosphohexose isomerase"[Title/Abstract] OR "small breast epithelial mucin"[Title/Abstract] OR Survivin[Title/Abstract] OR "Tumor-associated glycoprotein72"[Title/Abstract] OR "Tissue Inhibitor of Metalloproteinases-1"[Title/Abstract] OR "Thymidine kinase"[Title/Abstract] OR "Tissue polypeptide antigen"[Title/Abstract] OR "Tissue polypeptide-specific antigen"[Title/Abstract] OR "Urokinase plasminogen activator"[Title/Abstract] OR "plasminogen activator inhibitor-1"[Title/Abstract] OR "Vascular Endothelial Growth Factor"[Title/Abstract] OR "E_selectin:endothelial selectin"[Title/Abstract] OR "P-selectin:platelet selectin"[Title/Abstract] OR "intracellular adhesion molecule 1"[Title/Abstract]) Sort by:

Best Match

#6 48052

Add

Search "Biomarkers, Tumor/blood"[Mesh] Sort by: Best Match

#28 22831

Add

Search "Early Detection of Cancer"[Mesh] Sort by: Best Match

#22 282232

Add

Search "Breast Neoplasms"[Mesh] Sort by: Best Match

#17 258965

Add

Search "breast cancer"[Title/Abstract] Sort by: Best Match

#11 163954

Add

Search (60 OR #6)([60 OR #6](#_ENREF_60))([60 OR #6](#_ENREF_60))([60 OR #6](#_ENREF_60))([60 OR #6](#_ENREF_60))([60 OR #6](#_ENREF_60))([60 OR #6](#_ENREF_60))([59 OR #6](#_ENREF_59)) Sort by: Best Match

#31 48371

Add

Search ("Cancer Screening"[Title/Abstract] OR "Cancer diagnosis"[Title/Abstract]) Sort by: Best Match

#32 61334

Add

Search ("Early Detection of Cancer"[Mesh]) OR (("Cancer Screening"[Title/Abstract] OR

"Cancer diagnosis"[Title/Abstract])) Sort by: Best Match

#36 354640

Add

Search ("Breast Neoplasms"[Mesh]) OR "breast cancer"[Title/Abstract] Sort by: Best Match

#40 432

Add

Search ((((#1 OR #6))) AND (("Early Detection of Cancer"[Mesh]) OR (("Cancer Screening"[Title/Abstract] OR "Cancer diagnosis"[Title/Abstract])))) AND (("Breast Neoplasms"[Mesh]) OR "breast cancer"[Title/Abstract]) Sort by: Best Match

Embase: 94

#8 147,885

'breast cancer mucin':ab,ti OR 'cancer antigen27.29':ab,ti OR 'cancer antigen15.3':ab,ti OR 'cancer antigen125':ab,ti OR 'cancer antigen549':ab,ti OR 'cancer associated serum antigen':ab,ti OR 'catapsin d':ab,ti OR 'carcinoma embryonic antigen':ab,ti OR 'creatine kinase-brain':ab,ti OR 'carboxypeptidase n':ab,ti OR 'collapsin response mediator proteins':ab,ti OR 'colony stimulating factor1':ab,ti OR 'cytokeratin fragment':ab,ti OR 'galectin.3':ab,ti OR 'human epidermal growth factor receptor-2':ab,ti OR 'human mammaglobin':ab,ti OR 'mannose receptor':ab,ti OR 'mucin- like carcinoma':ab,ti OR 'matrix metalloproteinase-9':ab,ti OR 'mammary serum antigen':ab,ti OR 'nicotinamide phosphoribosyl transferase':ab,ti OR 'p53 protein':ab,ti OR 'phosphohexose isomerase':ab,ti OR 'small breast epithelial mucin':ab,ti OR survivin:ab,ti OR 'tumor-associated glycoprotein72':ab,ti OR 'tissue inhibitor of metalloproteinases-1':ab,ti OR 'thymidine kinase':ab,ti OR 'tissue polypeptide antigen':ab,ti OR 'tissue polypeptide-specific antigen':ab,ti OR 'urokinase plasminogen activator':ab,ti OR 'plasminogen activator inhibitor-1':ab,ti OR 'vascular endothelial growth factor':ab,ti OR 'e_selectin:endothelial selectin':ab,ti OR 'p-selectin:platelet selectin':ab,ti OR 'intracellular adhesion molecule 1':ab,ti

#9 535,315*

'breast tumor'/exp

#10 378,559*

'breast cancer':ab,ti OR 'breast tumor':ab,ti

#11 182,572*

'screening':kw OR 'diagnosis':kw OR 'early detection':kw

#12 573,058*

#9 OR #10

#13 94*

#8 AND #11 AND #12

Scopus: 1064

( ( TITLE-ABS-KEY ( "breast cancer mucin" OR "cancer antigen27.29" OR "cancer antigen15.3" OR "cancer antigen125" OR "cancer antigen549" OR "cancer associated serum antigen" OR "catapsin d" OR "carcinoma embryonic antigen" OR "creatine kinase-brain" OR "carboxypeptidase n" ) ) OR ( TITLE-ABS-KEY ( "collapsin response mediator proteins" OR "colony stimulating factor1" OR "cytokeratin fragment" OR "Galectin.3" OR "human epidermal growth factor receptor-2" OR "Human mammaglobin" ) ) OR ( TITLE-ABS-KEY ( "mannose receptor" OR "mucin- like carcinoma" OR "Matrix metalloproteinase-9" OR "Mammary serum antigen" OR "Nicotinamide phosphoribosyl transferase" OR "p53 protein" ) ) OR ( TITLE-ABS-KEY ( "phosphohexose isomerase" OR "small breast epithelial mucin" OR survivin OR "Tumor-associated glycoprotein72" OR "Tissue Inhibitor of Metalloproteinases-1" OR "Thymidine kinase" OR "Tissue polypeptide antigen" OR "Tissue polypeptide-specific antigen" ) ) OR ( TITLE-ABS-KEY ( "Urokinase plasminogen activator" OR "plasminogen activator inhibitor-1" OR "Vascular Endothelial Growth Factor" ) ) OR ( TITLE-ABS-KEY ( "E_selectin:endothelial selectin" OR "P-selectin:platelet selectin" OR "intracellular adhesion molecule 1" ) ) ) AND ( KEY ( "Breast Neoplasms" OR "breast cancer" ) ) AND ( KEY ( "Early Detection" OR "Early Diagnosis" OR screening ) )

Cochrane:53

#1 "breast cancer mucin" OR "cancer antigen27.29" OR "cancer antigen15.3" OR "cancer antigen125" OR "cancer antigen549" OR "cancer associated serum antigen" OR "catapsin d" OR "carcinoma embryonic antigen" OR "creatine kinase-brain" OR "carboxypeptidase n" OR "collapsin response mediator proteins" OR "colony stimulating factor1" OR "cytokeratin fragment" OR "Galectin.3" OR "human epidermal growth factor receptor-2" OR "Human mammaglobin" OR "mannose receptor" OR "mucin- like carcinoma" OR "Matrix metalloproteinase-9" OR "Mammary serum antigen" OR "Nicotinamide phosphoribosyl transferase" OR "p53 protein" OR "phosphohexose isomerase" OR "small breast epithelial mucin" OR Survivin OR "Tumor-associated glycoprotein72" OR "Tissue Inhibitor of Metalloproteinases-1" OR "Thymidine kinase" OR "Tissue polypeptide antigen" OR "Tissue polypeptide-specific antigen" OR "Urokinase plasminogen activator" OR "plasminogen activator inhibitor-1" OR "Vascular Endothelial Growth Factor" OR "E_selectin:endothelial selectin" OR "P-selectin:platelet selectin" OR "intracellular adhesion molecule 1"

#2 MeSH descriptor: [Early Detection of Cancer] explode all trees

#3 (screening OR diagnosis OR "early detection"):ti,ab,kw

#4 MeSH descriptor: [Breast Neoplasms] explode all trees

#5 ("breast cancer" OR "breast Tumor"):ti,ab,kw

#6 #2 OR #3

#7 #4 Or #5

#8 #1 AND #6 AND #7

**Appendix C:** Non-English articles and inaccessible articles.

1. Eskelinen, M., et al. (1988). "A new tumor marker MCA in breast cancer diagnosis." Anticancer Res **8**(4): 665-668.
2. Patel, P. S., et al. (1990). "Evaluation of serum sialic acid, heat stable alkaline phosphatase and fucose as markers of breast carcinoma." Anticancer Res 10(4): 1071-1074.
3. von Broen, B. and P. Kavungu (1990). "[Value of neopterin as an oncologic laboratory parameter--observations in gynecologic tumors]." Z Gesamte Inn Med 45(8): 223-225.
4. Astion, M. L. and P. Wilding (1992). "Application of neural networks to the interpretation of laboratory data in cancer diagnosis." Clin Chem 38(1): 34-38.
5. Shakhtarin, V. V., et al. (1992). "[Tumor markers MCA and CA-125 in the diagnosis and monitoring of breast cancer]." Vopr Onkol 38(4): 433-439.
6. Eskelinen, M., et al. (1994). "Clinical value of serum tumour markers TPA, TPS, TAG 12, CA 15-3 and MCA in breast cancer diagnosis; results from a prospective study." Anticancer Res 14(2b): 699-703.
7. Fontana, X., et al. (1994). "C-erb-B2 gene amplification and serum level of c-erb-B2 oncoprotein at primary breast cancer diagnosis." Anticancer Res 14(5b): 2099-2104.
8. Kiluk, M. S., et al. (2002). "[Usefulness of CEA, CA 15-3 and CA 125 tumor markers in the differential diagnostics of peritoneal effusion]." Pol Merkur Lekarski 13(76): 298-301.
9. Zheng, H. and R. C. Luo (2005). "[Diagnostic value of combined detection of TPS, CA153 and CEA in breast cancer]." Di Yi Jun Yi Da Xue Xue Bao 25(10): 1293-1294, 1298.
10. Chourin, S., et al. (2008). "[Routine use of serial plasmatic CA 15-3 determinations during the follow-up of patients treated for breast cancer.
11. Begley, S. (2009). "The myth of early detection." Newsweek 153(14): 44.
12. Watson, A. P. and K. A. Egland (2010). "Pathways to personalized medicine for breast and prostate cancers: emerging diagnostic methods and prognostic biomarkers." S D Med 63(7): 247-253.
13. Kopczynski, Z. and A. Thielemann (1998). "The value of tissue polypeptide specific antigen TPS determination in serum of women with breast cancer comparison to mucin-like associated antigen MCA and CA 15-3 antigen." Eur J Gynaecol Oncol 19(5): 503-507.
14. Eskelinen, M., et al. (1992). "A prospective study of tissue polypeptide specific antigen (TPS) in breast cancer diagnosis." Anticancer Research 12(6 B): 2033-2036.
15. Donadeo, A., et al. (1995). "MCA performance in preoperative breast cancer patients." Anticancer Research 15(2): 527-529.
16. Hare, W. S. C., et al. (1988). "Comparison of mammary serum antigen assay with mammography in patients with breast cancer." Medical Journal of Australia 149(8): 402-406.
17. Björklund, B. and R. Einarsson (1996). "TPS (tissue polypeptide specific antigen) in oncologic practice: A review with reference to 3000 cases of breast cancer." Tumor Diagnostik und Therapie 17(3): 67-73.
18. Oremek, G. M., et al. (1996). "Diagnostic value of mammary serum antigen-MSA as tumormarker for breast cancer." Medizinische Welt 47(11): 463-466.
19. Yasasever, V., et al. (1997). "Evaluation of phosphohexose isomerase as a metastasis marker in breast cancer patients." European Journal of Gynaecological Oncology 18(5): 397-399.
20. Einarsson, R. and V. Barak (1999). "TPS(TM): A cytokeratin serum tumor marker for effective of cancer patients with focus on breast cancer." Journal of Clinical Ligand Assay 22(4): 348-351.
21. Braden, A. M., et al. (2014). "Breast cancer biomarkers: Risk assessment, diagnosis, prognosis, prediction of treatment efficacy and toxicity, and recurrence." Current Pharmaceutical Design 20(30): 4879-4898.
22. Song, Y., et al. (2014). "Determination of expression of PAI-1 in sera of patients with breast cancer by antibody microarray." Chinese Journal of Biologicals 27(9): 1192-1196.
23. Mai, R., et al. (1991). "THE DIAGNOSTIC EFFICIENCY OF MUCIN-LIKE CARCINOMA ASSOCIATED ANTIGEN (MCA) IN BREAST-CANCER COMPARED TO CA-15-3."
24. Lamerz, R., et al. (1991). "ROLE OF BLOOD MARKERS IN THE DETECTION OF METASTASES FROM PRIMARY BREAST-CANCER." Diagnostic Oncology 1(2): 88-97.
25. Bjorklund, B. and R. Einarsson (1996). "TPS (tissue polypeptide specific antigen) in oncologic practice: A review with reference to 3000 cases of breast cancer."
26. Desmetz, C., et al. (2011). "[Serum autoantibodies profiling and early-stage cancer detection]." Med Sci (Paris) 27(6-7): 633-638.

**Appendix D:**

**Table 1: Fifty-four studies were identified relevant to our research question.**

| Method of chemical measurement | Number of panel components | panel | title | First author | country | year |
| --- | --- | --- | --- | --- | --- | --- |
| SELDI-TOF | 4 | BC1a/BC1b/BC3a/BC3b | Serum biomarkers for detection of breast cancers: A prospective study | Mathelin, C. | France. | 2006  1 |
| SELDI-TOF and MALDI-TOF-TOF | 4 | 6630 Da  +  9427 Da  +  6429 Da  +  6577 Da | Serum proteome profiling of primary breast cancer indicates a specific biomarker profile | Bohm, D. | Germany | 2011  2 |
| SELDI with immobilized  metal affinity ProteinChip Arrays | 3 | BC1, BC2, BC3 | Proteomics and bioinformatics approaches for identification of serum biomarkers to detect breast cancer | Li, Jinong | usa | 2002  3 |
| SELDI ProteinChip® mass spectrometry | 3(Using Strong Anion Exchange Chip Surface)  4(Using Strong Anion Exchange and Immobilized Copper Chip Surfaces) | 2.95 kd  +  3.68 kd  +  4.27 kd  **-**  2.95 kd  +  3.68 kd  +  4.27 kd  +  4.03 kd | A novel approach toward development of a rapid blood test for breast cancer | Vlahou, Antonia | usa | 2003  4 |
| SELDI-TOF-MS ProteinChip | 4 | CA1 (17.3 kDa)  +  CA2 (26.2 kDa)  +  CA3 (5.7 kDa)  +  CA4 (8.9 kDa) | SELDI-TOF-MS: the proteomics and bioinformatics approaches in the diagnosis of breast cancer | Hu, Yue | china | 2005  5 |
| MALDI-TOF mass spectrometer | 2 | Two first peaks | MALDI-TOF serum protein profiling for the detection of breast cancer | de Noo, Mirre E | Netherland | 2006  6 |
| MALDI-ToF mass spectrometry  MALDI-ToF mass spectrometry  +  ELISA | 3  4 | 3579 Da  +  2866  Da  +  2303  Da  and  3579 Da  +  2866  Da  +  2303  Da  +osteopontin | Mass spectrometry-based serum proteome pattern analysis in molecular diagnostics of early stage breast cancer | Pietrowska, Monika | Poland | 2009  7 |
| SELDITOF-MS | 3 | 6.630  Da(Apolipoprotein C-I)  +  8.139  Da(C-terminal-truncated form of C3a)  +  8.942 Da(Complement component C3a) | Detection and identification of potential biomarkers of breast cancer | Fan, Yuxia | China | 2010  8 |

| ELISA  CMIA | 2  2  3 | VEGF + CA 15-3  M-CSF + CA 15-3  VEGF + M-CSF + CA 15-3 | Diagnostic Power of Vascular Endothelial Growth Factor and Macrophage Colony-Stimulating Factor in Breast Cancer Patients Based on ROC Analysis | S Zajkowska, M.. | Poland | 2016  9 |
| --- | --- | --- | --- | --- | --- | --- |
| ELISA | 2 | MSA + CA15-3 | Comparison of mammary serum antigen (MSA) and CA15-3 levels in the serum of patients with breast cancer | Sacks, N. P. | Australia. | 1987  10 |
| ELISA | 2 | Free psa+psa ACT | Serum total and free prostate-specific antigen for breast cancer diagnosis in women | Black, M. H. | Canada. | 2000  11 |
| ELISA | 2 | FN1+ CXCL9 | Gene expression profiling identifies Fibronectin 1 and CXCL9 as candidate biomarkers for breast cancer screening | Ruiz-Garcia, E. | France | 2010  12 |
| Chemiluminescence Imaging Immunoassay | 3 | Ca15.3  +ca125  +cea | Chemiluminescence imaging immunoassay of multiple tumor markers for cancer screening | Zong, C. | China | 2012  13 |
| ELISA | 3 | TF1+TF2+TF3 | Serum TFF1 and TFF3 but not TFF2 are higher in women with breast cancer than in women without breast cancer | Ikemura, M. | Japan | 2017  14 |
| ELISA | 2 | MMP-9/TIMP-1  (ratio) | Clinical implications of proteolytic activity imbalance in breast cancer diagnosis | Swellam, M. | Egypt | 2014  15 |
| antibody-array platform | 4 | CSF2+TFRC+ RYBP+ ITGB4 | Candidate early detection protein biomarkers for ER+/PR+ invasive ductal breast carcinoma identified using pre-clinical plasma from the WHI observational study | Buas, M. F. | USA | 2015  16 |
| MRM based mass spectrometry | 3 | CHL1  +  APOC1  +  CA1 | Development and Validation of a Novel Plasma Protein Signature for Breast Cancer Diagnosis by Using Multiple Reaction Monitoring-based Mass Spectrometry | Lee, H. B. | Republic of Korea | 2015  17 |
| ELISA | 2  2  2 | PP2R4+Sclerostin  PPP2R4+IL-2  PPP2R4+CEA | Construction and analysis of the NCI-EDRN breast cancer reference set for circulating markers of disease | Marks, J. R. | usa | 2015  18 |

| ELISA | 2  2  2  3 | CA15-3 and CEA  VN and CA15-3  VN and CEA  VN and CA15-3 and CEA | Vitronectin: a promising breast cancer serum biomarker for early diagnosis of breast cancer in patients | Hao, W. | China | 2016  19 |
| --- | --- | --- | --- | --- | --- | --- |
| ELISA | 2 | FST and KLK6 | An integrated cell line-based discovery strategy identified follistatin and kallikrein 6 as serum biomarker candidates of breast carcinoma | Mange, A. | France | 2016  20 |
|  |  |  | Testing breast cancer serum biomarkers for early detection and prognosis in pre-diagnosis samples | Kazarian, A. | UK | 2017  21 |
| ELISA | 4 | p16 c-myc TP53 ANXA-1 | A panel of autoantibodies as potential early diagnostic serum biomarkers in patients with breast cancer | Liu, Y. | China | 2017  22 |
| Commercial kits | 5 | NSE  +  CA15.3  +  NGAL  +  EGFR  +  8-OHdG | Analysis of blood markers for early breast cancer diagnosis | Bayo, J. | Spain | 2018  23 |
| ELISA | 2  2  3 | Ca15.3  +  Cea  Cea+cerb-2  Ca15.3  +  Cea  +  Cerb-2 | c-erbB-2 oncoprotein, CEA, and CA 15.3 in patients with breast cancer: prognostic value | Molina, Rafael | spain | 1998  24 |
| ELISA | 3 | c-myc  +  Koc  +  cyclin B1  **sequential** | Recursive partitioning as an approach to selection of immune markers for tumor diagnosis | Koziol, James A | usa | 2003  25 |
| ELISA | 2 | anti-livin antibody  +  anti-survivin antibody | Detection of autoantibodies to survivin and livin in sera from patients with breast cancer | Yagihashi, Atsuhito | Japan | 2005  26 |

| ELISA | 3 | p16  +  c-myc  +  P53 | Humoral immune response to p16, a cyclin-dependent kinase inhibitor in human malignancies | Looi, Koksun | Usa.china | 2006  27 |
| --- | --- | --- | --- | --- | --- | --- |
|  |  |  |  |  |  |  |
| ELISA | 3 | ASB-9, SERAC1 and RELT | Autoantibodies as potential biomarkers for breast cancer | Zhong, Li | Usa.china | 2008  28 |
| ELISA | 5 | FKBP52 PPIA  PRDX2 HSP60 MUC1 | Identification of a new panel of serum autoantibodies associated with the presence of in situ carcinoma of the breast in younger women | Desmetz, Caroline | France | 2009  29 |
| ELISA | 3 | Mif  +  Mmp.9  +  mpo | Do serum biomarkers really measure breast cancer? | Jesneck, Jonathan L | usa | 2009  30 |
| ELISA | 2 | Ca15.3  +  ALCAM | Activated leukocyte cell adhesion molecule: a novel biomarker for breast cancer | Kulasingam, Vathany | Canada | 2009  31 |
| LC-MS/MS | 2 | ITIH4  +  Des-Arg9-bradykinin | Serum degradome markers for the detection of breast cancer | van Winden | Netherland | 2010  32 |
| ELISA | 4  3 | HER2 + p53 + CEA + cycB1  HER2 + p53 + cycB1 | Evaluation of known oncoantibodies, HER2, p53, and cyclin B1, in prediagnostic breast cancer sera | Lu, Hailing | usa | 2012  33 |
| ELISA | 2  2  2  3 | FTH1 + hnRnPF  FTH1 + CA 15-3  hnRnPF + CA 15-3  FTH1 + hnRnPF + CA 15-3 | Combined measurement of CA 15-3 with novel autoantibodies improves diagnostic accuracy for breast cancer | Dong, Xuejun | china | 2013  34 |
| **Discovery population set**  2de.gel  **Second set**  ELISA | 5 | GAL3, RACK1, PAK2, PHB2 and RUVBL1 | Identification and validation of new autoantibodies for the diagnosis of DCIS and node negative early‐stage breast cancers | Lacombe, Jérôme | france | 2013  35 |
| ELISA | 2  3  4  5 | c-myc  +  Surviving  c-myc+survivin+cyclin B1  c-myc+survivin+cyclin B1+cyclin D1  c-myc+survivin+cyclin B1+cyclin D1 +p62 | Mini-array of multiple tumor-associated antigens (TAAs) in the immunodiagnosis of breast cancer | Ye, HUA | Usa-china | 2013  36 |

| ELISA | 2 | EMA/CK1 ratio | Circulating levels and clinical implications of epithelial membrane antigen and cytokeratin-1 in women with breast cancer: can their ratio improve the results? | Attallah, Abdelfattah M | Egypt | 2014  37 |
| --- | --- | --- | --- | --- | --- | --- |

| ELISA | 2  3  4  5 | Cyclin B1+Imp1  Cyclin B1+Imp1+Koc  Cyclin B1+Imp1+Koc+survivin  Cyclin B1+Imp1+Koc+survivin+p16 | Detection of autoantibodies to multiple tumor-associated antigens (TAAs) in the immunodiagnosis of breast cancer | Liu, Weihong | China-usa | 2015  38 |
| --- | --- | --- | --- | --- | --- | --- |
| ELISA | 2  2  2  3  3  4 | MMP-9 + ca 15-3  TiMP-1 + ca 15-3  VegF + ca 15-3  VegF + MMP-9 + ca 15-3  VegF + TiMP-1 + ca 15-3  VegF + MMP-9 + TiMP-1 + ca 15-3 | Plasma levels and diagnostic utility of VEGF, MMP-9, and TIMP-1 in the diagnosis of patients with breast cancer | Ławicki, Sławomir | Poland | 2016  39 |
| ELISA | 2 | BCM-EIA and CAM 26 | SERUM MARKERS FOR PRIMARY AND RECURRENT BREAST-CANCER - BCM-EIA, CAM-26 AND CAM-29 | Ricketts, D. | uk | 1992  40 |
| ELISA | 2  2  2  3 | MMP-2+ CA 15-3  TIMP-2+ CA 15-3  VEGF+ CA 15-3  TIMP-2+ VEGF+ CA 15-3 | Plasma levels and diagnostic utility of VEGF, MMP-2 and TIMP-2 in the diagnostics of breast cancer patients | Lawicki, S. | poland | 2017  41 |
| Radioimmunoassay  +  ELISA | 2 | MSA + B2m | Comparison of mammary serum antigen (MSA) with β2-microglobulin (β2M) and carcinoembryonic antigen (CEA) assays in patients with breast cancer | Tjandra, JJ | australia | 1988  42 |
| ELISA | 2  2  2 | CA-15.3 +TPA  CA-15.3 +MCA  MCA +TPA | CA-15.3, TPA and MCA as markers for breast cancer | Barak, M | Israel | 1990  43 |
| ELISA | 2 | MMP-9/NGAL  Ratio | Circulating levels of matrix metalloproteinase-9 (MMP-9), neutrophil gelatinase-associated lipocalin (NGAL) and their complex MMP-9/NGAL in breast cancer disease | Provatopoulou, Xeni | Greece finland | 2009  44 |
| ELISA | 2  2  2  2  2 | IL-3+CA15-3  SCF+CA15-3  GM-CSF+CA15-3  G-CSF+CA15-3  M-CSF+CA15-3 | Hematopoietic cytokines as tumor markers in breast malignancies. A multivariate analysis with ROC curve in breast cancer patients | Ławicki, S | poland | 2013  45 |
| ELISA | 2  2  2  3 | VEGF+ CA 15-3  M-CSF+ CA 15-3  VEGF+ M-CSF  VEGF+ M-CSF+ CA 15-3 | VEGF, M-CSF and CA 15-3 as a new tumor marker panel in breast malignancies: a multivariate analysis with ROC curve | Ławicki, Sławomir | poland | 2013  46 |
| ELISA | 2 | CRP+ suPAR | Inflammatory biomarkers and cancer: CRP and suPAR as markers of incident cancer in patients with serious nonspecific symptoms and signs of cancer | Rasmussen, L. | Denmark | 2017  47 |
| ELISA | 2 | GATA3+E-cadherin | Evaluation of the value of GATA3 combined with E-cadherin in the diagnosis of breast cancer | Luo, M. | China | 2019  48 |
| ELISA | 2 | CASA  +  CA15.3 | CA15-3, CASA, MSA, and TPS as diagnostic serum markers in breast cancer | Devine, P. L. | Australia | 1995  49 |

|  | 4 | PDGF+ PF4+VEGF +TGF-𝛽 | Expression of angiogenesis regulatory proteins and epithelial-mesenchymal transition factors in platelets of the breast cancer patients | Han, H. | China | 2014  50 |
| --- | --- | --- | --- | --- | --- | --- |
| RIA kit | 2 | CEA  +  CA 15.3 | A re-evaluation of carcinoembryonic antigen (CEA) as a serum marker for breast cancer: a prospective longitudinal study | Guadagni, Fiorella | Italy | 2001  51 |
| ELISA  +  chemiluminescence | 2  2 | T1 vs healthy  TK 210 ELISA + CA15-3  T2 vs healthy  TK 210 ELISA + CA15-3 | A clinical evaluation of the TK 210 ELISA in sera from breast cancer patients demonstrates high sensitivity and specificity in all stages of disease | Kumar, J Kiran | Sweden | 2016  52 |
| MEIA  +  ELISA  +  **colorimetric method** | 2  2 | CA 15.3 + IL-18  CA 15.3 + NO | Significance of vascular endothelial growth factor, interleukin-18 and nitric oxide in patients with breast cancer: correlation with carbohydrate antigen 15.3 | Metwally, Fatehya M | Egypt | 2011  53 |

| ELISA | 4  4 | Antip53  +  Fn1  +  Ctgf  +  Wfdc2  Antip53  +  Fn1  +  Ca125  +  Wfdc2 | Evaluating Serum Markers for Hormone Receptor-Negative Breast Cancer | Schummer, M. | usa | 2015  54 |
| --- | --- | --- | --- | --- | --- | --- |

**Appendix E**

1. The list of multi-studies panels:

1. **M-CSF+CA15-3**

2. **VegF + ca 15-3**

3. **VEGF + M-CSF + CA 15-3**

4. **VEGF+ M-CSF**

5. **p16+ c-myc+ P53**

6. **CA15-3 +CEA**

2. The list of selected single study panels:

1. **MMP-9/TIMP-1**

2. **CA 15.3 + IL-18**

3. **MSA + B2m**

4. **GATA3+E-cadherin**

5. **TF1+TF2+TF3**

6. **MSA + CA15-3**

**Appendix F**

**
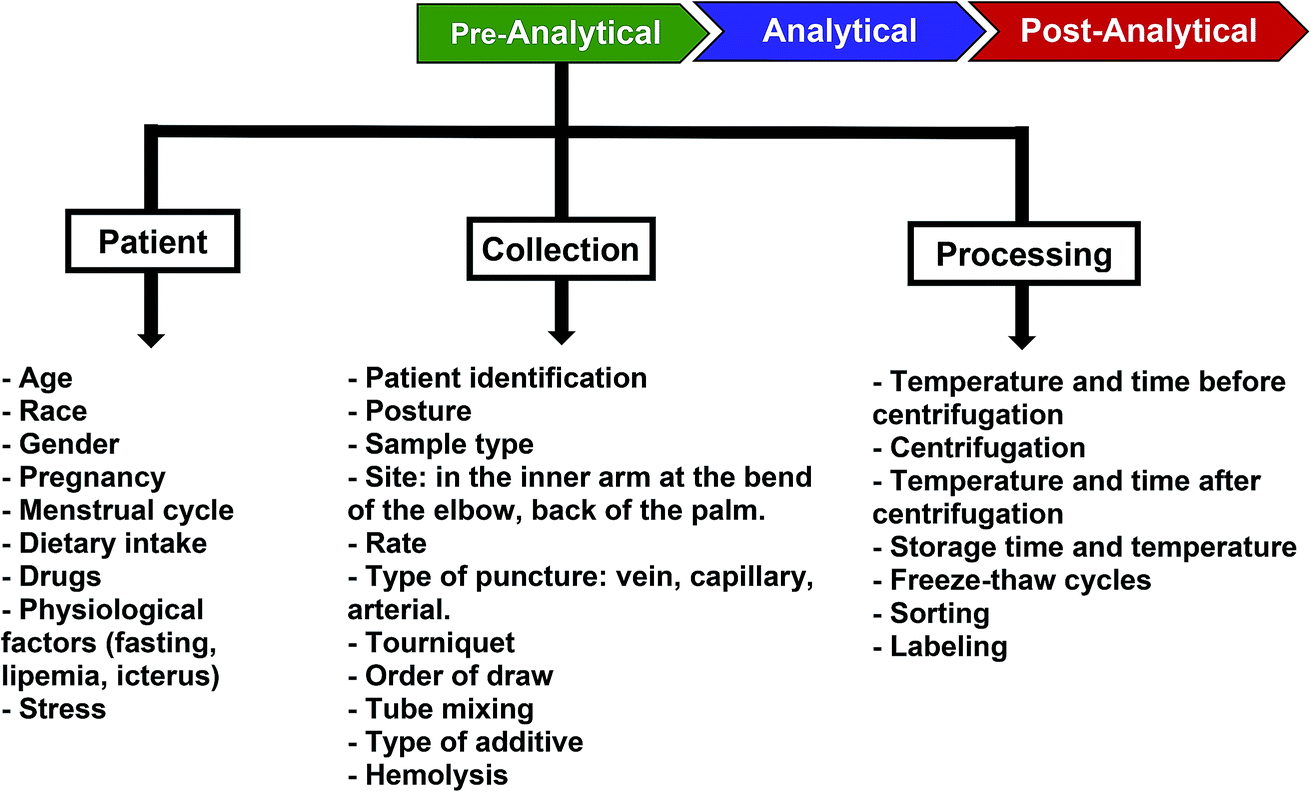
**

https://pubs.rsc.org/en/content/articlelanding/2019/ay/c9ay00131j#!divAbstract
